# Supplementary material for: Prime editing efficiently generates W542L and S621I double mutations in two ALS genes in maize
Source: Genome Biol. 2020 Oct 6;21:257. doi: 10.1186/s13059-020-02170-5 (PMC7541250; doi:10.1186/s13059-020-02170-5)
Supplement: Supplementary file 1 — Additional file 1: Figure S1. Schematic diagram of forming the two types of byproducts. Figure S2. Sequencing chromatograms from 7 prime-edited lines harboring W542L edits. Figure S3. Sequencing chromatograms from 2 prime-edited lines harboring homozygous S621I edits. Figure S4. Prime-editing efficiency in rice protoplasts for pegRNAs based on different expression strategies. Table S1. Edits and byproducts revealed from cloned PCR fragments. Table S2. Analysis of mutations in T0 transgenic plants by NGS with a 0.5% threshold. Table S3. Edits and byproducts from the 4 additional lines. Table S4. Prime-editing efficiency in rice protoplasts analyzed by NGS. Table S5. Sequences of primers, targets, and rtT-PBS of the pegRNAs. Supplemental material. Sequences of the PE2 and pegRNA expression cassettes. [file 13059_2020_2170_MOESM1_ESM.pdf]

Additional file 1

Table of contents

Figure S1. Schematic diagram for forming the two types of byproducts 3

Figure S2. Sequencing chromatograms from 7 prime-edited lines harboring W542L edits 4

Figure S3. Sequencing chromatograms from 2 prime-edited lines 5

Figure S4. Prime-editing efficiencies in rice protoplasts for pegRNAs based on different expression strategies 6

Table S1. Edits and byproducts revealed from cloned PCR fragments 7

Table S2. Analysis of mutations in T0 transgenic plants by NGS with 0.5% threshold 9

Table S3. Edits and byproducts from the 4 additional lines 11

Table S4. Prime-editing efficiency in rice protoplasts analyzed by NGS 12

Table S5. Sequences of primers, targets, and rtT-PBS of pegRNAs 13

Supplemental material. Sequences of PE2 and pegRNA expression cassettes 15

- Maize codon-optimized PE2 15
- Synthetic P165S-1 for generation of pZ1PE3b 16
- Synthetic P165S-2 for generation of pZ1PE3 16
- Synthetic W542L for generation of pG3R2R3-W542L (pZ1WS/pZ1WS-Csy4) 16
- Synthetic WS-Csy4 for generation of pL2L1-WS-Csy4 (pZ1WS-Csy4) 16
- Synthetic WS-pegR for generation of pL2L1-WS-pegR (pZ1WS) 17
- Synthetic WS-sgR for generation of pR1R4-WS-sgR (pZ1WS/pZ1WS-Csy4) 17
- Synthetic S621I for generation of pL4L3-S621I (pZ1WS/pZ1WS-Csy4) 17
- Synthetic OsALS-1pegR for generation of p35C-ALS-S1 and pU3-ALS-S1 17
- Synthetic OsGAPDH-1pegR for generation of p35C-GAPDH and pU3-GAPDH 17
- Synthetic OsALS-2pegR1 for generation of p35C-ALS-WS and pL2L1-ALS-WS 17

Synthetic OsALS-2pegR2 for generation of p2xU3-ALS-WS, pG3R23-OsWS, pR1R4-OsWS, and pL4L3-OsWS 18

The pegRNA and sgRNA cassettes in pZ1PE3, pZ1PE3b, and pZ1WS et al. 18

The two pegRNA cassettes in pL2L1-WS-Csy4 and pZ1WS-Csy4 18

The two pegRNA cassettes in pL2L1-WS-pegR and pZ1WS 19

The two sgRNA cassettes in pR1R4-WS-sgR, pZ1WS, and pZ1WS-Csy4 19

The pegRNA cassettes in pU3-ALS-S1 and pU3-GAPDH 19

The pegRNA cassettes in p35C-ALS-S1 and p35C-GAPDH 20

The pegRNA cassettes in p2xU3-ALS-WS, pG3R23-OsWS, pR1R4-OsWS, and pL4L3-OsWS 20

The pegRNA cassettes in p35C-ALS-WS and pL2L1-OsWS 20

Figure S1. Schematic diagram for forming the two types of byproducts

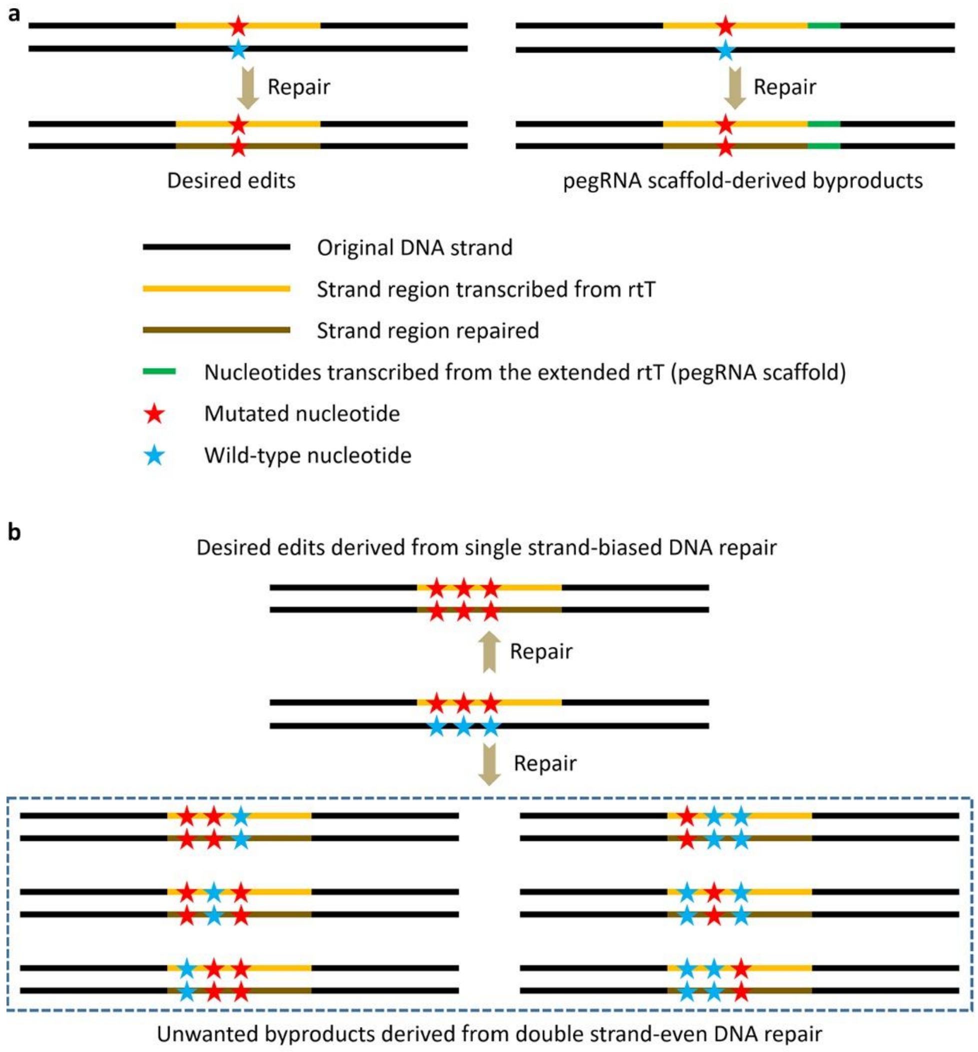

Fig. S1. Schematic diagram of forming the two types of byproducts. a Schematic diagram of forming the pegRNA scaffold-derived byproducts. **b** Schematic diagram of forming the double strand-even DNA repair byproducts.

Figure S2. Sequencing chromatograms from 7 prime-edited lines harboring W542L edits

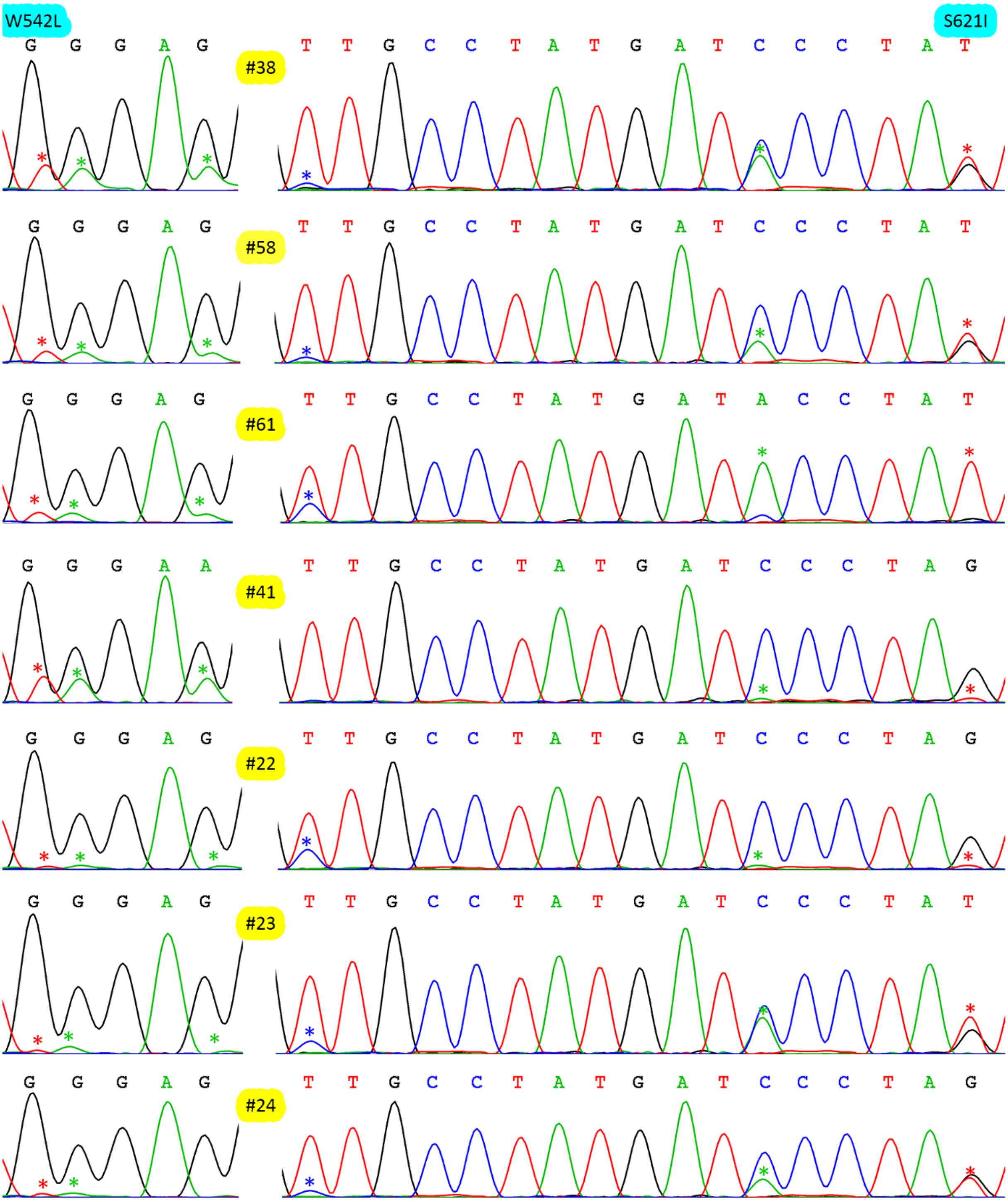

Fig. S2. Sequencing chromatograms from 7 prime-edited lines harboring W542L edits. Double peaks represent heterozygous or chimeric mutations and an asterisk indicates a mutation induced by PE. Note that the first asterisk of the S621I edits indicates the pegRNA scaffold-derived byproducts.

Figure S3. Sequencing chromatograms from 2 prime-edited lines

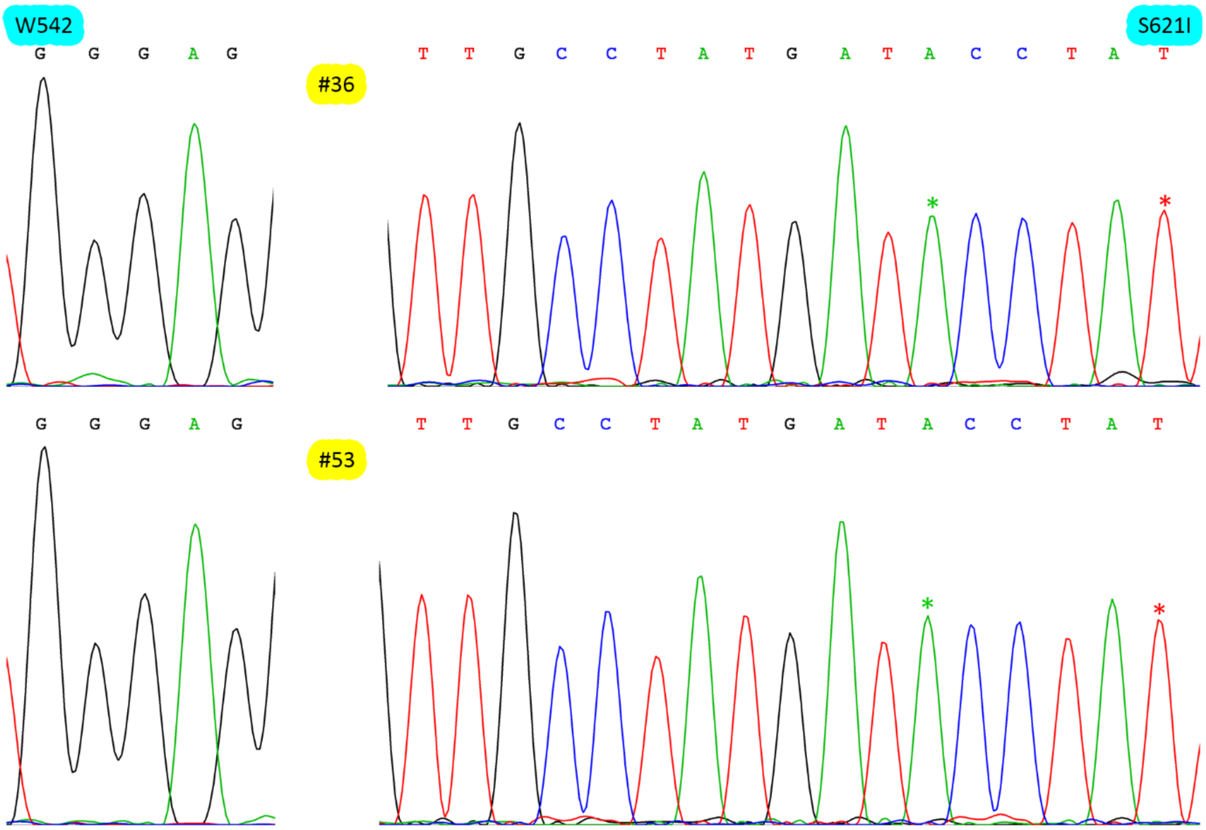

Fig. S3. Sequencing chromatograms from 2 prime-edited lines harboring homozygous S621I edits. An asterisk indicates a mutation induced by PE.

Figure S4. Prime-editing efficiencies in rice protoplasts for pegRNAs based on different expression strategies

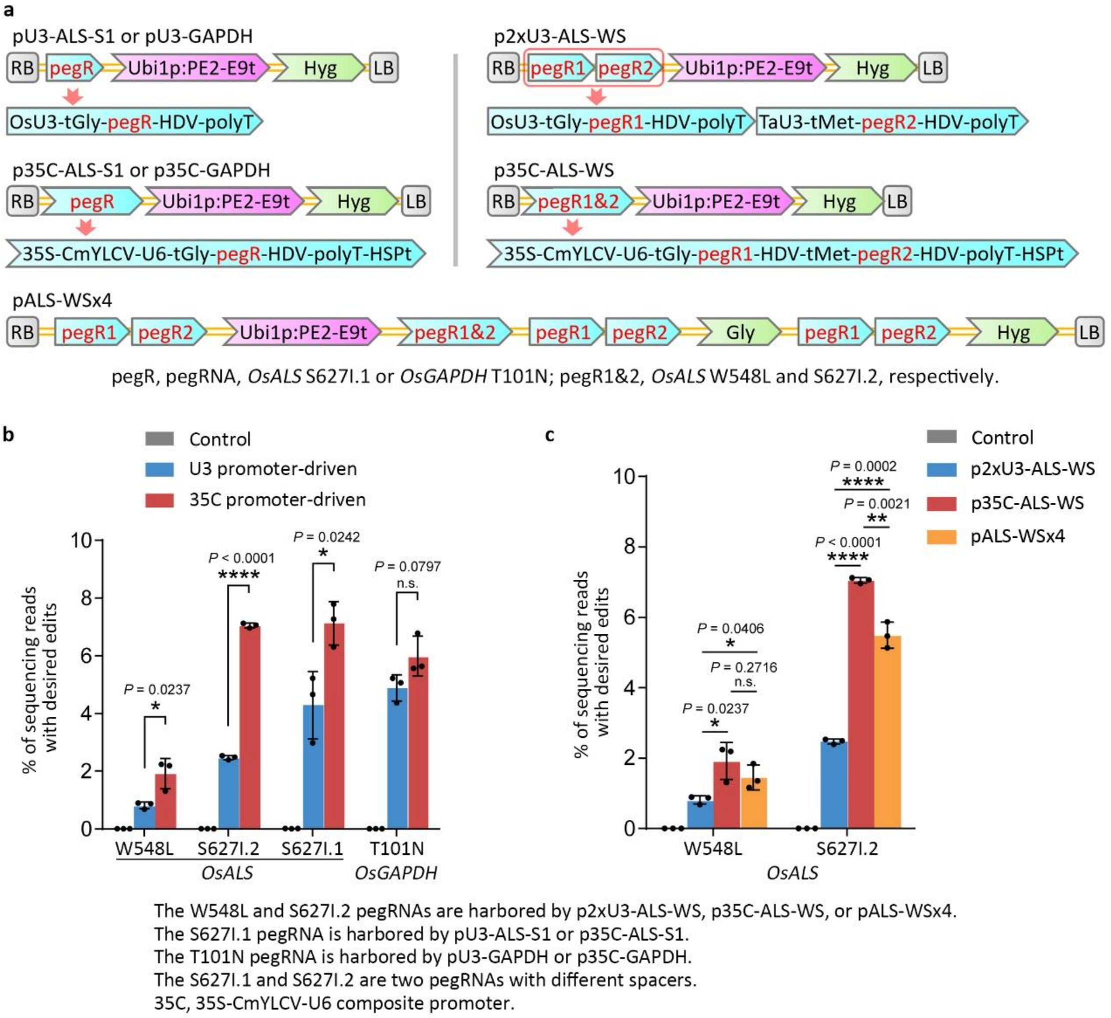

Fig. S4. Prime-editing efficiencies in rice protoplasts for pegRNAs based on different expression strategies. a T-DNA structures of the seven rice PE vectors. **b** Prime-editing efficiencies in rice protoplasts for pegRNAs driven by the 35S-CmYLCV-U6 composite promoter. An untreated protoplast sample served as a control. **c** Prime-editing efficiencies in rice protoplasts for pegRNAs based on the expression strategy of redoubling the number of expression cassettes. Efficiency (mean ± s.e.m.) was calculated from three independent experiments (n = 3). *P* values were obtained using the two-tailed Student's *t*-test. *$P < 0.05$, **$P < 0.01$, ***$P < 0.001$, ****$P < 0.0001$. n.s., $P > 0.05$.

Table S1. Edits and byproducts revealed from cloned PCR fragments

Table S1. Edits and byproducts revealed from cloned PCR fragments

Mutation	Line	Gene	Total	De	Sc	Re	To	De%	Sc%	Re%	To%	De/To	Sc/To	Re/To
**S621I**	**#3**	***ALS1***	48	1	0	2	3	2.1%	0	4.2%	6.3%	33.3%	0	66.7%
		ALS2	43	3	0	0	3	7.0%	0	0	7.0%	100%	0	0
		ALS1/2	91	4	0	2	6	4.4%	0	2.2%	6.6%	66.7%	0	33.3%
	**#4**	***ALS1***	41	41	0	0	41	100%	0	0	100%	100%	0	0
		ALS2	47	47	0	0	47	100%	0	0	100%	100%	0	0
		ALS1/2	88	88	0	0	88	100%	0	0	100%	100%	0	0
	**#5**	***ALS1***	51	7	0	1	8	13.7%	0	2.0%	15.7%	87.5%	0	12.5%
		ALS2	27	5	0	1	6	18.5%	0	3.7%	22.2%	83.3%	0	16.7%
		ALS1/2	78	12	0	2	14	15.4%	0	2.6%	17.9%	85.7%	0	14.3%
	**#6**	***ALS1***	40	3	1	0	4	7.5%	2.5%	0	10.0%	75.0%	25.0%	0
		ALS2	46	5	0	1	6	10.9%	0	2.2%	13.0%	83.3%	0	16.7%
		ALS1/2	86	8	1	1	10	9.3%	1.2%	1.2%	11.6%	80.0%	10.0%	10.0%
	**#8**	***ALS1***	48	6	1	1	8	12.5%	2.1%	2.1%	16.7%	75.0%	12.5%	12.5%
		ALS2	45	2	4	1	7	4.4%	8.9%	2.2%	15.6%	28.6%	57.1%	14.3%
		ALS1/2	93	8	5	2	15	8.6%	5.4%	2.2%	16.1%	53.3%	33.3%	13.3%
	**#14**	***ALS1***	45	7	0	1	8	15.6%	0	2.2%	17.8%	87.5%	0	12.5%
		ALS2	43	4	0	1	5	9.3%	0	2.3%	11.6%	80.0%	0	20.0%
		ALS1/2	88	11	0	2	13	12.5%	0	2.3%	14.8%	84.6%	0	15.4%
	**#15**	***ALS1***	56	12	36	3	51	21.4%	64.3%	5.4%	91.1%	23.5%	70.6%	5.9%
		ALS2	21	6	4	2	12	28.6%	19.0%	9.5%	57.1%	50.0%	33.3%	16.7%
		ALS1/2	77	18	40	5	63	23.4%	51.9%	6.5%	81.8%	28.6%	63.5%	7.9%
	**#16**	***ALS1***	54	3	4	2	9	5.6%	7.4%	3.7%	16.7%	33.3%	44.4%	22.2%
		ALS2	30	2	3	0	5	6.7%	10.0%	0	16.7%	40.0%	60.0%	0
		ALS1/2	84	5	7	2	14	6.0%	8.3%	2.4%	16.7%	35.7%	50.0%	14.3%
	**All-1**	***ALS1***	383	80	42	10	132	20.9%	11.0%	2.6%	34.5%	60.6%	31.8%	7.6%
		ALS2	302	74	11	6	91	24.5%	3.6%	2.0%	30.1%	81.3%	12.1%	6.6%
		ALS1/2	685	154	53	16	223	22.5%	7.7%	2.3%	32.6%	69.1%	23.8%	7.2%
	**All-2**	***ALS1***	286	27	6	7	40	9.4%	2.1%	2.4%	14.0%	67.5%	15.0%	17.5%
		ALS2	234	21	7	4	32	9.0%	3.0%	1.7%	13.7%	65.6%	21.9%	12.5%
		ALS1/2	520	48	13	11	72	9.2%	2.5%	2.1%	13.8%	66.7%	18.1%	15.3%
**W542L**	**#3**	***ALS1***	48	0	0	0	0	0	0	0	0	/	/	/
		ALS2	43	0	0	0	0	0	0	0	0	/	/	/
		ALS1/2	91	0	0	0	0	0	0	0	0	/	/	/
	**#4**	***ALS1***	41	0	0	0	0	0	0	0	0	/	/	/
		ALS2	47	0	0	0	0	0	0	0	0	/	/	/
		ALS1/2	88	0	0	0	0	0	0	0	0	/	/	/
	**#5**	***ALS1***	51	2	0	0	2	3.9%	0	0	3.9%	100%	0	0
		ALS2	27	1	0	0	1	3.7%	0	0	3.7%	100%	0	0
		ALS1/2	78	3	0	0	3	3.8%	0	0	3.8%	100%	0	0
	**#6**	***ALS1***	40	0	0	1	1	0	0	2.5%	2.5%	0	0	100%
		ALS2	46	1	0	0	1	2.2%	0	0	2.2%	100%	0	0
		ALS1/2	86	1	0	1	2	1.2%	0	1.2%	2.3%	50.0%	0	50.0%
	**#8**	***ALS1***	48	0	0	0	0	0	0	0	0	/	/	/
		ALS2	45	0	0	0	0	0	0	0	0	/	/	/
		ALS1/2	93	0	0	0	0	0	0	0	0	/	/	/
	**#14**	***ALS1***	45	0	0	0	0	0	0	0	0	/	/	/
		ALS2	43	0	0	0	0	0	0	0	0	/	/	/
		ALS1/2	88	0	0	0	0	0	0	0	0	/	/	/
	**#15**	***ALS1***	56	1	0	0	1	1.8%	0	0	1.8%	100%	0	0
		ALS2	21	0	0	0	0	0	0	0	0	/	/	/

		ALS1/2	77	1	0	0	1	1.3%	0	0	1.3%	100%	0	0
	**#16**	***ALS1***	54	1	1	0	2	1.9%	1.9%	0	3.7%	50.0%	50	0
		ALS2	30	1	0	0	1	3.3%	0	0	3.3%	100%	0	0
		ALS1/2	84	2	1	0	3	2.4%	1.2%	0	3.6%	66.7%	33.3%	0
	**All-1**	***ALS1***	383	4	1	1	6	1.0%	0.3%	0.3%	1.6%	66.7%	16.7%	16.7%
		ALS2	302	3	0	0	3	1.0%	0	0	1.0%	100%	0	0
		ALS1/2	685	7	1	1	9	1.0%	0.1%	0.1%	1.3%	77.8%	11.1%	11.1%
**P165S (PE3b)**	**#51**	***ALS1***	46	0	0	0	0	0	0	0	0	/	/	/
		ALS2	53	0	0	0	0	0	0	0	0	/	/	/
		ALS1/2	99	0	0	0	0	0	0	0	0	/	/	/
	**#161**	***ALS1***	36	0	0	0	0	0	0	0	0	/	/	/
		ALS2	25	0	0	0	0	0	0	0	0	/	/	/
		ALS1/2	61	0	0	0	0	0	0	0	0	/	/	/
	**#162**	***ALS1***	26	0	0	0	0	0	0	0	0	/	/	/
		ALS2	28	0	0	0	0	0	0	0	0	/	/	/
		ALS1/2	54	0	0	0	0	0	0	0	0	/	/	/
	**All-1**	***ALS1***	108	0	0	0	0	0	0	0	0	/	/	/
		ALS2	106	0	0	0	0	0	0	0	0	/	/	/
		ALS1/2	214	0	0	0	0	0	0	0	0	/	/	/
**P165S (PE3)**	**#54**	***ALS1***	33	0	0	0	0	0	0	0	0	/	/	/
		ALS2	31	0	0	0	0	0	0	0	0	/	/	/
		ALS1/2	64	0	0	0	0	0	0	0	0	/	/	/
	**#55**	***ALS1***	31	0	0	0	0	0	0	0	0	/	/	/
		ALS2	25	0	0	0	0	0	0	0	0	/	/	/
		ALS1/2	56	0	0	0	0	0	0	0	0	/	/	/
	**#56**	***ALS1***	41	0	0	0	0	0	0	0	0	/	/	/
		ALS2	34	0	0	0	0	0	0	0	0	/	/	/
		ALS1/2	75	0	0	0	0	0	0	0	0	/	/	/
	**All-1**	***ALS1***	105	0	0	0	0	0	0	0	0	/	/	/
		ALS2	90	0	0	0	0	0	0	0	0	/	/	/
		ALS1/2	195	0	0	0	0	0	0	0	0	/	/	/

Total, total number of sequenced clones; De, Sc, Re, or To, No. of clones harboring specified mutations; De, desired edits; Sc, pegRNA scaffold-derived byproducts; Re, double-strand even DNA repair-derived byproducts; To, total No. of cloned fragments harboring all the three types of mutations; De%, Sc%, Re%, or To%, ratio of clones harboring specified mutations to total number of sequenced clones; All-1, value for all the lines; All-2, value for all the lines but #4 and #15.

Table S2. Analysis of mutations in T0 transgenic plants by NGS with 0.5% threshold

Table S2. Analysis of mutations in T0 transgenic plants by NGS with 0.5% threshold

Mutation	Line	Gene	De%	Sc%	Re%	To%	De/To	Sc/To	Re/To
S621I	#1	*ALS1*	4.2%	0.9%	0.9%	6.0%	70.8%	14.9%	14.4%
		ALS2	4.0%	0.9%	0.8%	5.7%	69.4%	15.8%	14.7%
		ALS1/2	4.1%	0.9%	0.8%	5.8%	70.1%	15.3%	14.5%
	#3	*ALS1*	3.7%	0.7%	0.7%	5.1%	72.9%	13.4%	13.8%
		ALS2	3.6%	0.8%	0.7%	5.1%	70.6%	16.0%	13.4%
		ALS1/2	3.7%	0.7%	0.7%	5.1%	71.7%	14.7%	13.6%
	#4	*ALS1*	100%	0	0	100%	100%	0	0
		ALS2	100%	0	0	100%	100%	0	0
		ALS1/2	100%	0	0	100%	100%	0	0
	#5	*ALS1*	9.4%	1.6%	1.8%	12.7%	73.8%	12.3%	13.9%
		ALS2	9.4%	1.6%	1.8%	12.8%	73.4%	12.4%	14.2%
		ALS1/2	9.4%	1.6%	1.8%	12.8%	73.6%	12.3%	14.1%
	#6	*ALS1*	8.0%	1.3%	2.3%	11.6%	68.9%	11.3%	19.8%
		ALS2	7.7%	1.7%	2.0%	11.4%	67.9%	14.6%	17.5%
		ALS1/2	7.9%	1.5%	2.1%	11.5%	68.4%	12.9%	18.7%
	#8	*ALS1*	12.6%	3.2%	2.6%	18.5%	68.3%	17.4%	14.3%
		ALS2	12.4%	3.2%	2.9%	18.5%	67.3%	17.3%	15.5%
		ALS1/2	12.5%	3.2%	2.7%	18.5%	67.8%	17.3%	14.9%
	#9	*ALS1*	4.0%	0.8%	1.2%	6.0%	66.9%	12.8%	20.3%
		ALS2	3.7%	0.9%	1.0%	5.6%	66.2%	16.1%	17.7%
		ALS1/2	3.9%	0.8%	1.1%	5.8%	66.6%	14.3%	19.1%
	#12	*ALS1*	1.6%	0	0	1.6%	100%	0	0
		ALS2	1.7%	0	0	1.7%	100%	0	0
		ALS1/2	1.7%	0	0	1.7%	100%	0	0
	#13	*ALS1*	3.1%	0	0	3.1%	100%	0	0
		ALS2	2.8%	0	0	2.8%	100%	0	0
		ALS1/2	2.9%	0	0	2.9%	100%	0	0
	#14	*ALS1*	14.5%	0.6%	4.8%	19.9%	72.9%	2.9%	24.2%
		ALS2	15.1%	0.7%	2.8%	18.6%	81.4%	3.5%	15.1%
		ALS1/2	14.8%	0.6%	3.8%	19.2%	77.0%	3.2%	19.8%
	#15	*ALS1*	21.3%	56.4%	5.3%	83.0%	25.7%	68.0%	6.3%
		ALS2	39.5%	15.1%	8.8%	63.4%	62.3%	23.9%	13.8%
		ALS1/2	30.4%	35.8%	7.0%	73.2%	41.6%	48.9%	9.6%
	#16	*ALS1*	11.6%	3.0%	3.3%	17.8%	64.8%	16.8%	18.4%
		ALS2	13.0%	2.9%	3.4%	19.3%	67.2%	15.2%	17.6%
		ALS1/2	12.3%	3.0%	3.3%	18.6%	66.1%	16.0%	17.9%
	Aver-1	*ALS1*	16.2%	5.7%	1.9%	23.8%	68.0%	24.0%	8.0%
		ALS2	17.7%	2.3%	2.0%	22.1%	80.4%	10.5%	9.1%
		ALS1/2	17.0%	4.0%	2.0%	22.9%	74.0%	17.5%	8.5%
	Aver-2	*ALS1*	7.3%	1.2%	1.8%	10.2%	71.1%	11.7%	17.2%
		ALS2	7.3%	1.3%	1.5%	10.2%	72.4%	12.5%	15.2%

		ALS1/2	7.3%	1.2%	1.6%	10.2%	71.7%	12.1%	16.2%
**W542L**	**#5**	***ALS1***	1.3%	0	0	1.3%	100%	0	0
		ALS2	1.0%	0	0	1.0%	100%	0	0
		ALS1/2	1.2%	0	0	1.2%	100%	0	0
	**#6**	***ALS1***	0.8%	0	0	0.8%	100%	0	0
		ALS2	0.5%	0	0	0.5%	100%	0	0
		ALS1/2	0.7%	0	0	0.7%	100%	0	0
	**#8**	***ALS1***	1.0%	0	0	1.0%	100%	0	0
		ALS2	0.9%	0	0	0.9%	100%	0	0
		ALS1/2	0.9%	0	0	0.9%	100%	0	0
	**#13**	***ALS1***	1.3%	0	0	1.3%	100%	0	0
		ALS2	0.6%	0	0	0.6%	100%	0	0
		ALS1/2	0.9%	0	0	0.9%	100%	0	0
	**#15**	***ALS1***	4.6%	0	0	4.6%	100%	0	0
		ALS2	2.9%	0	0	2.9%	100%	0	0
		ALS1/2	3.8%	0	0	3.8%	100%	0	0
	**#16**	***ALS1***	1.6%	0	0	1.6%	100%	0	0
		ALS2	1.6%	0	0	1.6%	100%	0	0
		ALS1/2	1.6%	0	0	1.6%	100%	0	0
	**Aver-1**	***ALS1***	1.8%	0	0	1.8%	100%	0	0
		ALS2	1.2%	0	0	1.2%	100%	0	0
		ALS1/2	1.5%	0	0	1.5%	100%	0	0

De, Sc, Re, or To, No. of clones harboring specified mutations; De, desired edits; Sc, pegRNA scaffold-derived byproducts; Re, double-strand even DNA repair-derived byproducts; To, total No. of cloned fragments harboring all the three types of mutations; De%, Sc%, Re%, or To%, ratio of clones harboring specified mutations to total number of NGS reads; Aver-1, average value for all the lines; Aver-2, average value for all the lines but #4 and #15.

Table S3. Edits and byproducts from the 4 additional lines

Table S3. Edits and byproducts from the 4 additional lines

Mutation	Line	Gene	Total	De	Sc	Re	To	De%	Sc%	Re%	To%	De/To	Sc/To	Re/To
**S621I**	**#38**	***ALS1***	52	12	3	2	17	23.1%	5.8%	3.8%	32.7%	70.6%	17.6%	11.8%
		ALS2	61	25	4	2	31	41.0%	6.6%	3.3%	50.8%	80.6%	12.9%	6.5%
		ALS1/2	113	37	7	4	48	32.7%	6.2%	3.5%	42.5%	77.1%	14.6%	8.3%
	**#41**	***ALS1***	62	4	1	1	6	6.5%	1.6%	1.6%	9.7%	66.7%	16.7%	16.7%
		ALS2	51	5	2	0	7	9.8%	3.9%	0	13.7%	71.4%	28.6%	0
		ALS1/2	113	9	3	1	13	8.0%	2.7%	0.9%	11.5%	69.2%	23.1%	7.7%
	**#58**	***ALS1***	47	6	1	14	21	12.8%	2.1%	29.8%	44.7%	28.6%	4.8%	66.7%
		ALS2	45	5	4	6	15	11.1%	8.9%	13.3%	33.3%	33.3%	26.7%	40.0%
		ALS1/2	92	11	5	20	36	12.0%	5.4%	21.7%	39.1%	30.6%	13.9%	55.6%
	**#61**	***ALS1***	49	47	2	0	49	95.9%	4.1%	0	100.0%	95.9%	4.1%	0
		ALS2	57	13	25	1	39	22.8%	43.9%	1.8%	68.4%	33.3%	64.1%	2.6%
		ALS1/2	106	60	27	1	88	56.6%	25.5%	0.9%	83.0%	68.2%	30.7%	1.1%
	**All**	***ALS1***	210	69	7	17	93	32.9%	3.3%	8.1%	44.3%	74.2%	7.5%	18.3%
		ALS2	214	48	35	9	92	22.4%	16.4%	4.2%	43.0%	52.2%	38.0%	9.8%
		ALS1/2	424	117	42	26	185	27.6%	9.9%	6.1%	43.6%	63.2%	22.7%	14.1%
**W542L**	**#38**	***ALS1***	52	4	0	0	4	7.7%	0	0	7.7%	100.0%	0	0
		ALS2	61	33	0	1	34	54.1%	0	1.6%	55.7%	97.1%	0.0%	2.9%
		ALS1/2	113	37	0	1	38	32.7%	0	0.9%	33.6%	97.4%	0.0%	2.6%
	**#41**	***ALS1***	62	0	0	0	0	0	0	0	0	/	/	/
		ALS2	51	31	0	0	31	60.8%	0	0	60.8%	100.0%	0	0
		ALS1/2	113	31	0	0	31	27.4%	0	0	27.4%	100.0%	0	0
	**#58**	***ALS1***	47	7	0	0	7	14.9%	0	0	14.9%	100.0%	0	0
		ALS2	45	6	0	1	7	13.3%	0	2.2%	15.6%	85.7%	0	14.3%
		ALS1/2	92	13	0	1	14	14.1%	0	1.1%	15.2%	92.9%	0	7.1%
	**#61**	***ALS1***	49	3	0	1	4	6.1%	0	2.0%	8.2%	75.0%	0	25.0%
		ALS2	57	9	0	4	13	15.8%	0	7.0%	22.8%	69.2%	0	30.8%
		ALS1/2	106	12	0	5	17	11.3%	0	4.7%	16.0%	70.6%	0	29.4%
	**All**	***ALS1***	210	14	0	1	15	6.7%	0	0.5%	7.1%	93.3%	0	6.7%
		ALS2	214	79	0	6	85	36.9%	0	2.8%	39.7%	92.9%	0	7.1%
		ALS1/2	424	93	0	7	100	21.9%	0	1.7%	23.6%	93.0%	0	7.0%

Total, total number of sequenced clones; De, Sc, Re, or To, No. of clones harboring specified mutations; De, desired edits; Sc, pegRNA scaffold-derived byproducts; Re, double-strand even DNA repair-derived byproducts; To, total No. of cloned fragments harboring all the three types of mutations; De%, Sc%, Re%, or To%, ratio of clones harboring specified mutations to total number of sequenced clones; All, value for all the lines.

Table S4. Prime-editing efficiency in rice protoplasts analyzed by NGS

Table S4. Prime-editing efficiency in rice protoplasts analyzed by NGS

**pegRNA/Mutation**	**Vector**	**Promoter**	**Sequencing reads with desired edits (%)**			
			n=1	n=2	n=3	Aver.
W548L	/	/	0.0	0.0	0.0	0.0
	p2xU3-ALS-WS	U3	0.7	0.9	0.9	0.8
	p35C-ALS-WS	35C	2.2	1.3	2.2	1.9
	pALS-WSx4	U3&35C	1.8	1.2	1.4	1.5
S627I.2	/	/	0.0	0.0	0.0	0.0
	p2xU3-ALS-WS	U3	2.5	2.4	2.5	2.5
	p35C-ALS-WS	35C	7.1	7.0	7.1	7.0
	pALS-WSx4	U3&35C	5.9	5.1	5.5	5.5
S627I.1	/	/	0.0	0.0	0.0	0.0
	pU3-ALS-S1	U3	3.0	5.2	4.7	4.3
	p35C-ALS-S1	35C	7.3	6.3	7.8	7.1
T101N	/	/	0.0	0.0	0.0	0.0
	pU3-GAPDH	U3	5.1	4.4	5.2	4.9
	p35C-GAPDH	35C	5.6	5.6	6.8	6.0

35C, CaMV35S-CmYLCV-U6 composite promoter. Aver., average value for three independent experiments (n = 1, 2, 3).

Table S5. Sequences of primers, targets, and rtT-PBS of pegRNAs

Table S5. Sequences of primers, targets, and rtT-PBS of pegRNAs

Sequence name	Sequence	Purpose
oHEASmE-F	AGCTTGCTGAATTCGTCAACACCTGCAACACTAGT	Vector construction
oHEASmE-R	AATTACTAGTGTTGCAGGTGTTGACGAATTCAGCA	
OsU3p-AsF	ATTTATTTAGGCGCGCCAGTAATTCATCCAGGTCAC	
TaU3t-EcR	AACACCATGAATTCAAGATGTTGTACTTCTGAA	
oiSce-HSF	AGCTTGAGACCATTACCCTGTTATCCCTAGGTCTCGAATTA	
oiSce-HSR	CTAGTAATTCGAGACCTAGGGATAACAGGGTAATGGTCTCA	
ALS1&2P-F	CGTCATCGCCAACCACCTCTTC	Analysis of the P165S mutation by Sanger sequencing
ALS1&2P-R	CCATCTGCTGCTGGATGTCCTTG	
ALS1&2WS-F	CTTGGGGCTATGGGATTTGGTTTGC	Analysis of the W542L and S621I mutations by Sanger sequencing
ALS1&2WS-R	TACACAGTCCTGCCATCACCATC	
ALS1&2WS-F2	CTTCTGTGGCCAACCCAGGTGT	
ALS1&2P-NGSF	GGAGTGAGTACGGTGTGCCGTCTGCATCGCCACCTC	Analysis of the P165S mutation by NGS
ALS1&2P-NGSR	GAGTTGGATGCTGGATGGACGATGGGCGTCTCCTG	
ALS1&2W-NGSF	GGAGTGAGTACGGTGTGCGATCCGAATTGAGAACCTCC	Analysis of the W542L mutation by NGS
ALS1&2W-NGSR	GAGTTGGATGCTGGATGGCATTCTCTGGGTTTCCCAAG	
ALS1&2S-NGSF	GGAGTGAGTACGGTGTGCAGGGCCGTACCTCTTGGATA	Analysis of the S621I mutation by NGS
ALS1&2S-NGSR	GAGTTGGATGCTGGATGGACAGTCCTGCCATCACCAT	
P35C-GAPDH-T101N/F	AGTTCCGGTAGCGAGCGTGGAACTGATG	Analysis of the T101N mutation of *OsGAPDH* by NGS
p35C-GAPDH-T101N/R	AGTCAACTTGCTTGTATGCAATCCCATGGG	
pU3-GAPDH-T101N/F	CCGTCCGGTAGCGAGCGTGGAACTGATG	
pU3-GAPDH-T101N/R	ATGTCACTTGCTTGTATGCAATCCCATGGG	
GAPDH-Control/F	CGTACGGGTAGCGAGCGTGGAACTGATG	
GAPDH-Control/R	GTTTCGCTTGCTTGTATGCAATCCCATGGG	
p2xU3-ALS-WS-W548L/F	CGATGTTTCAGGAGCTGGCATTGATC	Analysis of the W548L mutation of *OsALS* by NGS
p2xU3-ALS-WS-W548L/R	TGACCAAGCAATAGTCACAAAATCTGG	
p35C-ALS-WS-W548L/F	ACAGTGTTCAGGAGCTGGCATTGATC	
p35C-ALS-WS-W548L/R	GCCAATAGCAATAGTCACAAAATCTGG	
pALS-WSx4-W548L/F	CAGATCTTCAGGAGCTGGCATTGATC	
pALS-WSx4-W548L/R	CTTGTAAGCAATAGTCACAAAATCTGG	
ALS-W548L-Control/F	TAGCTTTTCAGGAGCTGGCATTGATC	
ALS-W548L-Control/R	GGCTACAGCAATAGTCACAAAATCTGG	
p2xU3-ALS-WS-S627I.2/F	CGATGTCCGCCATCAAGAAGATGC	Analysis of the S627I mutation of *OsALS* by NGS
p2xU3-ALS-WS-S627I.2/R	TGACCATTCAGGTCAAACATAGGCCG	
p35C-ALS-WS-S627I.2/F	ACAGTGCCGCCATCAAGAAGATGC	
p35C-ALS-WS-S627I.2/R	GCCAATTTCAGGTCAAACATAGGCCG	
pALS-WSx4-S627I.2/F	CAGATCCCGCCATCAAGAAGATGC	
pALS-WSx4-S627I.2/R	CTTGTATTCAGGTCAAACATAGGCCG	
p35C-ALS-S627I.1/F	ATCACGCCGCCATCAAGAAGATGC	
p35C-ALS-S627I.1/R	TTAGGCTTCAGGTCAAACATAGGCCG	
pU3-ALS-S627I.1/F	ACTTGACCGCCATCAAGAAGATGC	
pU3-ALS-S627I.1/R	GATCAGTTCAGGTCAAACATAGGCCG	

ALS-S627I-Control/F	TAGCTTCCGCCATCAAGAAGATGC	
ALS-S627I-Control/R	GGCTACTTCAGGTCAAACATAGGCCG	
ALS1&2P-T1	TCGGTGCCAATCATGCGTCGCGG	Two pegRNAs and two sgRNAs
P165S-rtT/PBS	GGACAGGTGAGTCGA/CGCATGATTG	for the P165S mutation
ALS1&2P-T2	CAGGTGAGTCGACGCATGATTGG	
P165S-rtT/PBS2	GGACAGGTGAGTCGA/CGCATGATTGGCA	
ALS1&2P-T2b	YCAGGAGACGCCCATCGTCGAGG (Y = T or C)	
ALS1&2W-T1	SGGGATGGTGGTGCAGTGGGAGG (S = C or G)	One pegRNA and one sgRNA
W542L-rtT/PBS	TAGAACCTGTCTTCTA/ACTGCACCACCAT	for the W542L mutation
ALS1W-T2	CTAACTGCACCACCATCCCGAGG	
ALS1&2S-T1	CCTTGAAAGCCCCACCACTAGGG	One pegRNA and one sgRNA
S621I-rtT/PBS	TGCCTATGATACCTAT/TGGTGGGGCTTTC	for the S621I mutation
ALS1&2S-T2	GATATGATCCTGGATGGTGATGG	
OsALS-S627I.1-T1	GTGCTGCCTATGATCCCAAGTGG	One pegRNA for the *OsALS*
OsALS-S627I.1-rtT/PBS	TGAATGCGCCCCCAaTT/GGGATCATAG	S627I mutation
OsGAPDH-T1	GAGTATGTCGTGGAGTCCACCGG	One pegRNA for the *OsGAPDH*
OsGAPDH-rtT/PBS	AGTGAAGACACCGtTG/GACTCCACGACA	T101N mutation
OsALS-W548L-T1	GGGTATGGTGGTGCAATGGGAGG	One pegRNA for the *OsALS*
OsALS-W548L-rtT/PBS	AAACCTATCtTCta/ATTGCACCACCAT	W548L mutation
OsALS-S627I.2-T1	CCTTGAATGCGCCCCCACTTGGG	Another pegRNA for the *OsALS*
OsALS-S627I.2-rtT/PBS	TGCCTATGATaCCAAt/TGGGGGCGCATTC	S627I mutation

Supplemental material. Sequences of PE2 and pegRNA expression cassettes

Maize codon-optimized PE2

NLS-SpCas9H840A-linker-M_MLV_RT-NLS

atgaagaggacagccgatggcagcgagttcgagagccctaagaagaagaggaaggtggacaagaagtactcgatcggcctcgatattgggactaactctgttggct
gggccgtgatcaccgacgagtacaaggtgccctcaaagaagttcaaggtcctgggcaacaccgatcggcattccatcaagaagaatctcattggcgctctcctgttcg
acagcggcgagacggctgaggctacgcggctcaagcgcaccgcccgcaggcggtacacgcgcaggaagaatcgcatctgctacctgcaggagattttctccaacga
gatggcgaaggttgacgattctttcttccacaggctggaggagtcattcctcgtggaggaggataagaagcacgagcggcatccaatcttcggcaacattgtcgacga
ggttgcctaccacgagaagtaccctacgatctaccatctgcggaagaagctcgtggactccacagataaggcggacctccgcctgatctacctcgctctggcccacat
gattaagttcaggggccatttcctgatcgagggggatctcaacccggacaatagcgatgttgacaagctgttcatccagctcgtgcagacgtacaaccagctcttcgag
gagaaccccattaatgcgtcaggcgtcgacgcgaaggctatcctgtccgctaggctctcgaagtctcggcgcctcgagaacctgatcgcccagctgccgggcgagaag
aagaacggcctgttcgggaatctcattgcgctcagcctggggctcacgcccaacttcaagtcgaatttcgatctcgctgaggacgccaagctgcagctctccaaggac
acatacgacgatgacctggataacctcctggcccagatcggcgatcagtacgcggacctgttcctcgctgccaagaatctgtcggacgccatcctcctgtctgatattct
cagggtgaacaccgagattacgaaggctccgctctcagcctccatgatcaagcgctacgacgagcaccatcaggatctgaccctcctgaaggcgctggtcaggcagc
agctccccgagaagtacaaggagatcttcttcgatcagtcgaagaacggctacgctgggtacattgacggcggggcctctcaggaggagttctacaagttcatcaagc
cgattctggagaagatggacggcacggaggagctgctggtgaagctcaatcgcgaggacctcctgaggaagcagcggacattcgataacggcagcatcccacacca
gattcatctcggggagctgcacgctatcctgaggaggcaggaggacttctaccctttcctcaaggataaccgcgagaagatcgagaagattctgactttcaggatcccg
tactacgtcggcccactcgctaggggcaactcccgcttcgcttggatgacccgcaagtcagaggagacgatcacgccgtggaacttcgaggaggtggtcgacaaggg
cgctagcgctcagtcgttcatcgagaggatgacgaatttcgacaagaacctgccaaatgagaaggtgctccctaagcactcgctcctgtacgagtacttcacagtctac
aacgagctgactaaggtgaagtatgtgaccgagggcatgaggaagccggctttcctgtctggggagcagaagaaggccatcgtggacctcctgttcaagaccaaccg
gaaggtcacggttaagcagctcaaggaggactacttcaagaagattgagtgcttcgattcggtcgagatctctggcgttgaggaccgcttcaacgcctccctggggacc
taccacgatctcctgaagatcattaaggataaggacttcctggacaacgaggagaatgaggatatcctcgaggacattgtgctgacactcactctgttcgaggaccgg
gagatgatcgaggagcgcctgaagacttacgcccatctcttcgatgacaaggtcatgaagcagctcaagaggaggaggtacaccggctggggggaggctgagcagga
agctcatcaacggcattcgggacaagcagtccgggaagacgatcctcgacttcctgaagagcgatggcttcgcgaaccgcaatttcatgcagctgattcacgatgaca
gcctcacattcaaggaggatatccagaaggctcaggtgagcggccaggggactcgctgcacgagcatatcgcgaacctcgctggctcgccagctatcaagaaggg
gattctgcagaccgtgaaggttgtggacgagctggtgaaggtcatgggcaggcacaagcctgagaacatcgtcattgagatggcccgggagaatcagaccacgcag
aagggccagaagaactcacgcgagaggatgaagaggatcgaggagggcattaaggagctggggtcccagatcctcaaggagcacccggtggagaacacgcagct
gcagaatgagaagctctacctgtactacctccagaatggccgcgatatgtatgtggaccaggagctggatattaacaggctcagcgattacgacgtcgatgccatcgtt
ccacagtcattcctgaaggatgactccattgacaacaaggtcctcaccaggtcggacaagaaccggggcaagtctgataatgttccttcagaggaggtcgttaagaag
atgaagaactactggcgccagctcctgaatgccaagctgatcacgcagcggaagttcgataacctcacaaaggctgagaggggcgggctctctgagctggacaagg
cgggcttcatcaagaggcagctggtcgagacacggcagatcactaagcacgttgcgcagattctcgactcacggatgaacactaagtacgatgagaatgacaagctg
atccgcgaggtgaaggtcatcaccctgaagtcaaagctcgtctccgacttcaggaaggatttccagttctacaaggttcgggagatcaacaattaccaccatgcccatg
acgcgtacctgaacgcggtggtcggcacagctctgatcaagaagtacccaaagctcgagagcgagttcgtgtacggggactacaaggtttacgatgtgaggaagatg
atcgccaagtcggagcaggagattggcaaggctaccgccaagtacttcttctactctaacattatgaatttcttcaagacagagatcactctggccaatggcgagatcc
ggaagcgccccctcatcgagacgaacggcgagacgggggagatcgtgtgggacaagggcagggatttcgcgaccgtcaggaaggttctctccatgccacaagtgaa
tatcgtcaagaagacagaggtccagactggcgggttctctaaggagtcaattctgcctaagcggaacagcgacaagctcatcgcccgcaagaaggactgggatccga
agaagtacggcgggttcgacagccccactgtggcctactcggtcctggttgtggcgaaggttgagaagggcaagtccaagaagctcaagagcgtgaaggagctgct
ggggatcacgattatggagcgctccagcttcgagaagaacccgatcgatttcctggaggcgaagggctacaaggaggtgaagaaggacctgatcattaagctcccca
agtactcactcttcgagctggagaacggcaggaagcggatgctggcttccgctggcgagctgcagaaggggaacgagctggctctgccgtccaagtatgtgaacttcc
tctacctggcctcccactacgagaagctcaagggcagccccgaggacaacgagcagaagcagctgttcgtcgagcagcacaagcattacctcgacgagatcattgag
cagatttccgagttctccaagcgcgtgatcctggccgacgcgaatctggataaggtcctctccgcgtacaacaagcaccgcgacaagccaatcagggagcaggctga
gaatatcattcatctcttcaccctgacgaacctcggcgcccctgctgctttcaagtacttcgacacaactatcgatcgcaagaggtacacaagcactaaggaggtcctg
gacgcgaccctcatccaccagtcgattaccggcctctacgagacgcgcatcgacctgtctcagctcgggggcgactcaggcggctcatcgggcgggtcaagcgggtc
ggagacaccgggcacatcagagagcgctacccctgagtcatcaggcggctcttcaggcggcagctcaaccctgaacattgaggacgagtaccggctgcacgagacg
agcaaggagccagacgtttcgctcggcagcacttggctctctgacttcccacaggcttgggccgagactggcggcatgggcctggccgtgcgccaggctccactgatc
atccctctgaaggcgacctccaccccggtttctattaagcagtacccgatgagccaggaggccaggctggggatcaagccacacattcagcggctgctggaccaggg
catcctggtgccatgccagtccccgtggaatactccgctcctgccggtgaagaagcctgggacaaacgactacaggccggttcaggatctcagggaggtgaacaagc
gcgtggaggacatccatccgacagtgccgaacccgtacaatctgctgtcgggcctgcctccgagccaccagtggtacaccgtcctggacctcaaggacgctttcttctg
cctgcggctgcacccgacgtctcagccgctgttcgcgttcgagtggcgcgacccagagatgggcatttccggccagctgacctggacacgcctaccccagggcttcaa
gaactccccgactctcttcaacgaggctctccaccgggatctcgcggacttcaggattcagcatcccgatctgatcctgctccagtatgttgacgacctcctcctggccgc
gacgtcggagctggactgccagcagggcacccgggcgctgctgcagacactgggcaatctggggtaccgcgcctctgcgaagaaggcgcagatctgccagaagca
agtgaagtacctgggctacctcctgaaggagggccagcgctggctcactgaggcgaggaaggagactgttatgggccagcccactccaaagactccgaggcagctc
agggagttcctcggcaaggctgggttctgccgcctgttcatccctgggttcgctgagatggctgcgccgctctacccgctgactaagccggggacactgttcaactggg

ggccagaccagcagaaggcgtaccaggagattaagcaggcgctgctgacggccccagcgctcggcctaccagacctgacgaagccgttcgagctgttcgttgacga
gaagcaggggtacgcgaagggcgtgctgacacagaagctggggccttggcgccgcccggtcgcgtacctgtcgaagaagctggacccagtcgctgctgggtggcct
ccatgcctccggatggtcgctgctattgcggttctgaccaaggatgcggggaagctcacaatggggcagcctctcgtgatcctggctccacatgcggtggaggcgctg
gtgaagcagccaccggaccggtggctgtcgaacgctcggatgacacactaccaggcgctcctcctcgatacagaccgggttcagttcgggcctgtggttgctctgaac
ccagccacactgctgccactccctgaggagggcctccagcacaattgcctcgacatcctggctgaggcgcacggcacccgccctgatctcaccgaccagcctctgcca
gatgctgaccacacctggtacacggatgggtcctcgctgctgcaggagggccagaggaaggcgggcgccgccgtcaccacagagacagaggttatttgggccaagg
ccctaccggctggcaccagcgcccagcgcgctgagctgatcgcgctgactcaggcgctgaagatggccgaggggaagaagctcaatgtttacaccgactcgcggtac
gcgttcgctacagctcacattcatggggagatctaccgccggcgcgggtggctgacttcggagggcaaggagattaagaataaggacgagatcctggccctgctcaa
ggcgctgttcctgccgaagcgcctctcaatcattcactgcccgggccaccagaagggccattcggccgaggctaggggcaatcggatggctgaccaggcggcgcgga
aggcggctatcaccgagactcccgatacatctaccctcctgatcgagaactcgagcccaagcggcgggagcaagcggactgcggatgggtctgagttcgagccaaa
gaagaagaggaaggtgtga

Synthetic P165S-1 for generation of pZ1PE3b

BsaI-T1P-sgR-rtT-PBS-OsU3t-TaU3p-T2P-BsaI

GGTCTCTGGCGCGGTGCCAATCATGCGTCGGTTTTAGAGCTAGAAATAGCAAGTTAAAATAAGGCTAGTCCGTTATCAACTTGAAAAAGTGGCACC
GAGTCGGTGCGGACAGGTGAGTCGACGCATGATTGTTTTTTTTTTCGTTTTGCATTGAGTTTTCTCCGTCGCATGTTTGCAGCATGAATCCAAACCACA
CGGAGTTCAAATTCCCACAGATTAAGGCTCGTCCGTCGCACAAGGTAATGTGTGAATATTATATCTGTCGTGCAAAATTGCCTGGCCTGCACAATTGCTG
TTATAGTTGGCGGCAGGGAGAGTTTTAACATTGACTAGCGTGCTGATAATTTGTGAGAAATAATAATTGACAAGTAGATACTGACATTTGAGAAGAGCT
TCTGAACTGTTATTAGTAACAAAAATGGAAAGCTGATGCACGGAAAAAGGAAAGAAAAAGCCATACTTTTTTTTAGGTAGGAAAAGAAAAAGCCATAC
GAGACTGATGTCTCTCAGATGGGCCGGGATCTGTCTATCTAGCAGGCAGCAGCCCACCAACCTCACGGGCCAGCAATTACGAGTCCTTCTAAAAGCTC
CCGCCGAGGGGCGCTGGCGCTGCTGTGCAGCAGCACGTCTAACATTAGTCCCACCTCGCCAGTTTACAGGGAGCAGAACCAGCTTATAAGCGGAGGC
GCGGCACCAAGAAGCGAGGTGAGTCGACGCATGATGTTTAGAGACC

Synthetic P165S-2 for generation of pZ1PE3

BsaI-T1P-sgR-rtT-PBS-OsU3t-TaU3p-T2P-BsaI

GGTCTCTGGCGCGGTGCCAATCATGCGTCGGTTTTAGAGCTAGAAATAGCAAGTTAAAATAAGGCTAGTCCGTTATCAACTTGAAAAAGTGGCACC
GAGTCGGTGCGGACAGGTGagtCGACGCATGATTGGCATTTTTTTTTTCGTTTTGCATTGAGTTTTCTCCGTCGCATGTTTGCAGCATGAATCCAAACC
ACACGGAGTTCAAATTCCCACAGATTAAGGCTCGTCCGTCGCACAAGGTAATGTGTGAATATTATATCTGTCGTGCAAAATTGCCTGGCCTGCACAATTG
CTGTTATAGTTGGCGGCAGGGAGAGTTTTAACATTGACTAGCGTGCTGATAATTTGTGAGAAATAATAATTGACAAGTAGATACTGACATTTGAGAAGA
GCTTCTGAACTGTTATTAGTAACAAAAATGGAAAGCTGATGCACGGAAAAAGGAAAGAAAAAGCCATACTTTTTTTTAGGTAGGAAAAGAAAAAGCC
ATACGAGACTGATGTCTCTCAGATGGGCCGGGATCTGTCTATCTAGCAGGCAGCAGCCCACCAACCTCACGGGCCAGCAATTACGAGTCCTTCTAAAA
GCTCCCGCCGAGGGGCGCTGGCGCTGCTGTGCAGCAGCACGTCTAACATTAGTCCCACCTCGCCAGTTTACAGGGAGCAGAACCAGCTTATAAGCGG
AGGCGCGGCACCAAGAAGCGCAGGAGACGCCCATCGTCGGTTTAGAGACC

Synthetic W542L for generation of pG3R2R3-W542L (pZ1WS/pZ1WS-Csy4)

BsaI-T1W-sgR-rtT-PBS-OsU3t-TaU3p-T2W-BsaI

GGTCTCTGGCGGGGATGGTGGTGCAGTGGGGTTTTAGAGCTAGAAATAGCAAGTTAAAATAAGGCTAGTCCGTTATCAACTTGAAAAAGTGGCAC
CGAGTCGGTGCTAGAACCTGTCTTCTAACTGCACCACCATTTTTTTTTTTCGTTTTGCATTGAGTTTTCTCCGTCGCATGTTTGCAGCATGAATCCAAAC
CACACGGAGTTCAAATTCCCACAGATTAAGGCTCGTCCGTCGCACAAGGTAATGTGTGAATATTATATCTGTCGTGCAAAATTGCCTGGCCTGCACAATT
GCTGTTATAGTTGGCGGCAGGGAGAGTTTTAACATTGACTAGCGTGCTGATAATTTGTGAGAAATAATAATTGACAAGTAGATACTGACATTTGAGAAG
AGCTTCTGAACTGTTATTAGTAACAAAAATGGAAAGCTGATGCACGGAAAAAGGAAAGAAAAAGCCATACTTTTTTTTAGGTAGGAAAAGAAAAAGC
CATACGAGACTGATGTCTCTCAGATGGGCCGGGATCTGTCTATCTAGCAGGCAGCAGCCCACCAACCTCACGGGCCAGCAATTACGAGTCCTTCTAAAA
GCTCCCGCCGAGGGGCGCTGGCGCTGCTGTGCAGCAGCACGTCTAACATTAGTCCCACCTCGCCAGTTTACAGGGAGCAGAACCAGCTTATAAGCGG
AGGCGCGGCACCAAGAAGCGTAACTGCACCACCATCCCGGTTTAGAGACC

Synthetic WS-Csy4 for generation of pL2L1-WS-Csy4 (pZ1WS-Csy4)

BsaI-T1W-sgR-rtT-PBS-HDV-Csy4-T1S-sgR-rtT-PBS-BsaI

GGTCTCATGCACGGGATGGTGGTGCAGTGGGGTTTTAGAGCTAGAAATAGCAAGTTAAAATAAGGCTAGTCCGTTATCAACTTGAAAAAGTGGCA
CCGAGTCGGTGCTAGAACCTGTCTTCTAACTGCACCACCATGGCCGGCATGGTCCCAGCCTCCTCGCTGGCGCCGGCTGGGCAACATGCTTCGGCAT
GGCGAATGGGACGTTCACTGCCGTATAGGCAGCCTTGAAAGCCCCACCACTAGTTTTAGAGCTAGAAATAGCAAGTTAAAATAAGGCTAGTCCGTT
ATCAACTTGAAAAAGTGGCACCGAGTCGGTGCTGCCTATGATACCTATTGGTGGGGCTTTCGGCCAGAGACC

Synthetic WS-pegR for generation of pL2L1-WS-pegR (pZ1WS)

BsaI-T1W-sgR-rtT-PBS-HDV-tMet-T1S-sgR-rtT-PBS-BsaI

GGTCTCATGCACGGGATGGTGGTGCAGTGGGGTTTTAGAGCTAGAAATAGCAAGTTAAAATAAGGCTAGTCCGTTATCAACTTGAAAAAGTGGCA
CCGAGTCGGTGCTAGAACCTGTCTTCTAACTGCACCACCATGGCCGGCATGGTCCCAGCCTCCTCGCTGGCGCCGGCTGGGCAACATGCTTCGGCAT
GGCGAATGGGACAACAACAAATCAGAGTGGCGCAGCGGAAGCGTGGTGGGCCCATAACCCACAGGTCCCAGGATCGAAACCTGGCTCTGATACCT
TGAAAGCCCCACCACTAGTTTTAGAGCTAGAAATAGCAAGTTAAAATAAGGCTAGTCCGTTATCAACTTGAAAAAGTGGCACCGAGTCGGTGCTGC
CTATGATACCTATTGGTGGGGCTTTCGGCCAGAGACC

Synthetic WS-sgR for generation of pR1R4-WS-sgR (pZ1WS/pZ1WS-Csy4)

BsaI-T2W-sgR-OsU3t-TaU3p-T2S-BsaI

GGTCTCTGGCGTAACTGCACCACCATCCCGGTTTTAGAGCTAGAAATAGCAAGTTAAAATAAGGCTAGTCCGTTATCAACTTGAAAAAGTGGCACCG
AGTCGGTGCTTTTTTTTTTCGTTTTGCATTGAGTTTTCTCCGTCGCATGTTTGCAGCATGAATCCAAACCACACGGAGTTCAAATTCCCACAGATTAAGG
CTCGTCCGTCGCACAAGGTAATGTGTGAATATTATATCTGTCGTGCAAAATTGCCTGGCCTGCACAATTGCTGTTATAGTTGGCGGCAGGGAGAGTTTTA
ACATTGACTAGCGTGCTGATAATTTGTGAGAAATAATAATTGACAAGTAGATACTGACATTTGAGAAGAGCTTCTGAACTGTTATTAGTAACAAAAATGG
AAAGCTGATGCACGGAAAAAGGAAAGAAAAAGCCATACTTTTTTTTAGGTAGGAAAAGAAAAAGCCATACGAGACTGATGTCTCTCAGATGGGCCGG
GATCTGTCTATCTAGCAGGCAGCAGCCCACCAACCTCACGGGCCAGCAATTACGAGTCCTTCTAAAAGCTCCCGCCGAGGGGCGCTGGCGCTGCTGTG
CAGCAGCACGTCTAACATTAGTCCCACCTCGCCAGTTTACAGGGAGCAGAACCAGCTTATAAGCGGAGGCGCGGCACCAAGAAGCGATATGATCCTG
GATGGTGAGTTTAGAGACC

Synthetic S621I for generation of pL4L3-S621I (pZ1WS/pZ1WS-Csy4)

BsaI-T1S-sgR-rtT-PBS-OsU3t-TaU3p-T2S-BsaI

GGTCTCTGGCGCTTGAAAGCCCCACCACTAGTTTTAGAGCTAGAAATAGCAAGTTAAAATAAGGCTAGTCCGTTATCAACTTGAAAAAGTGGCACC
GAGTCGGTGCTGCCTATGATACCTATTGGTGGGGCTTTCTTTTTTTTTTCGTTTTGCATTGAGTTTTCTCCGTCGCATGTTTGCAGCATGAATCCAAACC
ACACGGAGTTCAAATTCCCACAGATTAAGGCTCGTCCGTCGCACAAGGTAATGTGTGAATATTATATCTGTCGTGCAAAATTGCCTGGCCTGCACAATTG
CTGTTATAGTTGGCGGCAGGGAGAGTTTTAACATTGACTAGCGTGCTGATAATTTGTGAGAAATAATAATTGACAAGTAGATACTGACATTTGAGAAGA
GCTTCTGAACTGTTATTAGTAACAAAAATGGAAAGCTGATGCACGGAAAAAGGAAAGAAAAAGCCATACTTTTTTTTAGGTAGGAAAAGAAAAAGCC
ATACGAGACTGATGTCTCTCAGATGGGCCGGGATCTGTCTATCTAGCAGGCAGCAGCCCACCAACCTCACGGGCCAGCAATTACGAGTCCTTCTAAAA
GCTCCCGCCGAGGGGCGCTGGCGCTGCTGTGCAGCAGCACGTCTAACATTAGTCCCACCTCGCCAGTTTACAGGGAGCAGAACCAGCTTATAAGCGG
AGGCGCGGCACCAAGAAGCGATATGATCCTGGATGGTGAGTTTAGAGACC

Synthetic OsALS-1pegR for generation of p35C-ALS-S1 and pU3-ALS-S1

BsaI-Spacer-sgR-rtT-PBS-BsaI

GGTCTCATGCAGTGCTGCCTATGATCCCAAGGTTTTAGAGCTAGAAATAGCAAGTTAAAATAAGGCTAGTCCGTTATCAACTTGAAAAAGTGGCACC
GAGTCGGTGCTGAATGCGCCCCCAaTTGGGATCATAGGGCCAGAGACC

Synthetic OsGAPDH-1pegR for generation of p35C-GAPDH and pU3-GAPDH

BsaI-Spacer-sgR-rtT-PBS-BsaI

GGTCTCATGCAGAGTATGTCGTGGAGTCCACGTTTTAGAGCTAGAAATAGCAAGTTAAAATAAGGCTAGTCCGTTATCAACTTGAAAAAGTGGCAC
CGAGTCGGTGCAGTGAAGACACCGtTGGACTCCACGACAGGCCAGAGACC

Synthetic OsALS-2pegR1 for generation of p35C-ALS-WS and pL2L1-ALS-WS

BsaI-Spacer-sgR-rtT-PBS-HDV-tMet-Spacer-sgR-rtT-PBS-BsaI

GGTCTCATGCAGGGTATGGTGGTGCAATGGGGTTTTAGAGCTAGAAATAGCAAGTTAAAATAAGGCTAGTCCGTTATCAACTTGAAAAAGTGGCAC
CGAGTCGGTGCAAACCTATCTTCTAATTGCACCACCATGGCCGGCATGGTCCCAGCCTCCTCGCTGGCGCCGGCTGGGCAACATGCTTCGGCATGG
CGAATGGGACAACAACAAATCAGAGTGGCGCAGCGGAAGCGTGGTGGGCCCATAACCCACAGGTCCCAGGATCGAAACCTGGCTCTGATACCTTG
AATGCGCCCCCACTTGTTTTAGAGCTAGAAATAGCAAGTTAAAATAAGGCTAGTCCGTTATCAACTTGAAAAAGTGGCACCGAGTCGGTGCTGCCTA
TGATACCAATTGGGGGCGCATTCGGCCAGAGACC

Synthetic OsALS-2pegR2 for generation of p2xU3-ALS-WS, pG3R23-OsWS, pR1R4-OsWS, and pL4L3-OsWS

BsaI-Spacer-sgR-rtT-PBS-HDV-OsU3t-TaU3p-tMet-Spacer-sgR-rtT-PBS-BsaI

GGTCTCATGCAGGGTATGGTGGTGCAATGGGGTTTTAGAGCTAGAAATAGCAAGTTAAAATAAGGCTAGTCCGTTATCAACTTGAAAAAGTGGCAC
CGAGTCGGTGCAAACCTATCTTCTAATTGCACCACCATGGCCGGCATGGTCCCAGCCTCCTCGCTGGCGCCGGCTGGGCAACATGCTTCGGCATGG
CGAATGGGACTTTTTTTTTTCGTTTTGCATTGAGTTTTCTCCGTCGCATGTTTGCAGTTTTATTTTCCGTTTTGCATTGAAATTTCTCCGTCTCATGTTTG
C**AG**CGTGTTCAACATGAATCCAAACCACACGGAGTTCAAATTCCCACAGATTAAGGCTCGTCCGTCGCACAAGGTAATGTGTGAATATTATATCTGT
CGTGCAAAATTGCCTGGCCTGCACAATTGCTGTTATAGTTGGCGGCAGGGAGAGTTTTAACATTGACTAGCGTGCTGATAATTTGTGAGAAATAAT
AATTGACAAGTAGATACTGACATTTGAGAAGAGCTTCTGAACTGTTATTAGTAACAAAAATGGAAAGCTGATGCACGGAAAAAGGAAAGAAAAAG
CCATACTTTTTTTTAGGTAGGAAAAGAAAAAGCCATACGAGACTGATGTCTCTCAGATGGGCCGGGATCTGTCTATCTAGCAGGCAGCAGCCCACC
AACCTCACGGGCCAGCAATTACGAGTCCTTCTAAAAGCTCCCGCCGAGGGGCGCTGGCGCTGCTGTGCAGCAGCACGTCTAACATTAGTCCCACCT
CGCCAGTTTACAGGGAGCAGAACCAGCTTATAAGCGGAGGCGCGGCACCAAGAAGCGAACAACAAAACAACAAATCAGAGTGGCGCAGCGGAAG
CGTGGTGGGCCCATAACCCACAGGTCCCAGGATCGAAACCTGGCTCTGATACCTTGAATGCGCCCCCACTTGTTTTAGAGCTAGAAATAGCAAGTTA
AAATAAGGCTAGTCCGTTATCAACTTGAAAAAGTGGCACCGAGTCGGTGCTGCCTATGATACCAATTGGGGGCGCATTCGGCCAGAGACC

The pegRNA and sgRNA cassettes in pZ1PE3, pZ1PE3b, and pZ1WS et al.

OsU3p-T1-sgR-rtT-PBS-OsU3t-TaU3p-T2-sgR-TaU3t

AGTAATTCATCCAGGTCACCAAGTTCTAGGATTTTCAGAACTGCAACTTATTTTATCAAGGAATCTTTAAACATACGAACAGATCACTTAAAGTTCTTCTG
AAGCAACTTAAAGTTATCAGGCTTGCATGGATCTTGGAGGAATCAGATGTGCAGTCAGGGACCATAGCACAAGACAGGCGTCTTCTACTGGTGCTACC
AGCAAATGCTGGAAGCCGGGAACACTGGGTACGTTGGAAACCACGTGATGTGAAGAAGTAAGATAAACTGTAGGAGAAAAGCATTTCGTAGTGGGC
CATGAAGCCTTTCAGGACATGTATTGCAGTATGGGCCGGCCCATTACGCAATTGGACGACAACAAAGTCTAGTATTAGTACCACCTCGGCTATCCACATA
GATCAAAGCTGATTTAAAAGAGTTGTGCAGATGATCCGTGGCGNNNNNNNNNNNNNNNNNNNGTTTTAGAGCTAGAAATAGCAAGTTAAAATAA
GGCTAGTCCGTTATCAACTTGAAAAAGTGGCACCGAGTCGGTGCNNNNNNNNNNNNNNNNNNNNNNNNNNNNNTTTTTTTTTTCGTTTTGCAT
TGAGTTTTCTCCGTCGCATGTTTGCAGCATGAATCCAAACCACACGGAGTTCAAATTCCCACAGATTAAGGCTCGTCCGTCGCACAAGGTAATGTGTGA
ATATTATATCTGTCGTGCAAAATTGCCTGGCCTGCACAATTGCTGTTATAGTTGGCGGCAGGGAGAGTTTTAACATTGACTAGCGTGCTGATAATTTGTG
AGAAATAATAATTGACAAGTAGATACTGACATTTGAGAAGAGCTTCTGAACTGTTATTAGTAACAAAAATGGAAAGCTGATGCACGGAAAAAGGAAAG
AAAAAGCCATACTTTTTTTTAGGTAGGAAAAGAAAAAGCCATACGAGACTGATGTCTCTCAGATGGGCCGGGATCTGTCTATCTAGCAGGCAGCAGCC
CACCAACCTCACGGGCCAGCAATTACGAGTCCTTCTAAAAGCTCCCGCCGAGGGGCGCTGGCGCTGCTGTGCAGCAGCACGTCTAACATTAGTCCCAC
CTCGCCAGTTTACAGGGAGCAGAACCAGCTTATAAGCGGAGGCGCGGCACCAAGAAGCGNNNNNNNNNNNNNNNNNNNGTTTTAGAGCTAGAA
ATAGCAAGTTAAAATAAGGCTAGTCCGTTATCAACTTGAAAAAGTGGCACCGAGTCGGTGCTTTTTTTTTTGTCCTTCTGTTTTTTTAGTCAGTCTCTTTT
TTCAGAAGTACAACATCTT

Note: the underlined part comes from the synthetic fragments and cassettes with the same structure as this one are harbored in pZ1PE3, pZ1PE3b, pG3R2R3-W542L, pL4L3-S621I, pZ1WS, and pZ1WS-Csy4.

The two pegRNA cassettes in pL2L1-WS-Csy4 and pZ1WS-Csy4

35S-CmYLCV-Csy4-P2A-ZsGreen-polyA-Csy4-T1W-sgR-rtT-PBS-HDV-Csy4-T1S-sgR-rtT-PBS-HDV-Csy4-HSPt

ATGGAGTCAAAGATTCAAATAGAGGACCTAACAGAACTCGCCGTAAAGACTGGCGAACAGTTCATACAGAGTCTCTTACGACTCAATGACAAGAAGAA
AATCTTCGTCAACATGGTGGAGCACGACACACTTGTCTACTCCAAAAATATCAAAGATACAGTCTCAGAAGACCAAAGGGCAATTGAGACTTTTCAACA
AAGGGTAATATCCGGAAACCTCCTCGGATTCCATTGCCCAGCTATCTGTCACTTTATTGTGAAGATAGTGGAAAAGGAAGGTGGCTCCTACAAATGCCA
TCATTGCGATAAAGGAAAGGCCATCGTTGAAGATGCCTCTGCCGACAGTGGTCCCAAAGATGGACCCCCACCCACGAGGAGCATCGTGGAAAAAGAA
GACGTTCCAACCACGTCTTCAAAGCAAGTGGATTGATGTGATTGGCAGACATACTGTCCCACAAATGAAGATGGAATCTGTAAAAGAAAACGCGTGAA
ATAATGCGTCTGACAAAGGTTAGGTCGGCTGCCTTTAATCAATACCAAAGTGGTCCCTACCACGATGGAAAAACTGTGCAGTCGGTTTGGCTTTTTCTG
ACGAACAAATAAGATTCGTGGCCGACAGGTGGGGGTCCACCATGTGAAGGCATCTTCAGACTCCAATAATGGAGCAATGACGTAAGGGCTTACGAAA
TAAGTAAGGGTAGTTTGGGAAATGTCCACTCACCCGTCAGTCTATAAATACTTAGCCCCTCCCTCATTGTTAAGGGAGCAAAATCTCAGAGAGATAGTCC
TAGAGAGAGAAAGAGAGCAAGTAGCCTAGAAGTAGTCAAGGCGGCGAAGTATTCAGGCACGTGGCCAGGAAGAAGAAAAGCCAAGACGACGAAA
ACAGGTAAGAGCTAAGCATCTAGATGGACCACTACCTGGACATCCGGCTCCGCCCGGACCCCGAGTTCCCGCCCGCCCAGCTGATGTCCGTGCTCTTC
GGCAAGCTGCACCAGGCCCTCGTGGCCCAGGGCGGCGACCGCATCGGCGTCTCCTTCCCGGACCTGGACGAGTCCAGGAGCCGCCTGGGCGAGAG
GCTCCGCATCCACGCCAGCGCGGACGACCTGAGGGCCCTCCTCGCCCGCCCGTGGCTGGAGGGCCTCAGGGACCACCTCCAGTTCGGCGAGCCGGC
CGTGGTCCCCCACCCAACCCCGTACCGCCAGGTGTCCCGCGTGCAGGCCAAGTCCAACCCCGAGAGGCTGCGCAGGCGCCTCATGAGGCGCCACGA
CCTGTCGGAGGAGGAGGCTAGGAAGCGCATCCCCGACACCGTGGCTAGGGCGCTGGACCTCCCCTTCGTCACCCTCCGCTCCCAGAGCACGGGCCA
GCACTTCCGGCTGTTCATCCGCCACGGCCCACTCCAGGTGACCGCCGAGGAGGGCGGCTTCACGTGCTACGGCCTCTCCAAGGGCGGCTTCGTCCCG
TGGTTCGGGCTCCGGCGCCACCAACTTCTCGCTGCTGAAGCAGGCGGGCGACGTGGAGGAGAACCCGGGCCCGATGGCCCAGTCCAAGCACGGCC
TGACCAAGGAGATGACGATGAAGTACCGCATGGAGGGCTGCGTGGACGGCCACAAGTTCGTCATCACCGGCGAGGGCATCGGCTACCCGTTCAAGG
GCAAGCAGGCCATCAACCTCTGCGTGGTGGAGGGCGGCCCCCTGCCGTTCGCCGAGGACATCCTCAGCGCCGCGTTCATGTACGGCAACCGCGTGTT
CACCGAGTACCCGCAGGACATCGTGGACTACTTCAAGAACTCCTGCCCAGCCGGCTACACGTGGGACAGGAGCTTCCTGTTCGAGGACGGCGCCGTG
TGCATCTGCAACGCGGACATCACCGTGTCCGTCGAGGAGAACTGCATGTACCACGAGAGCAAGTTCTACGGCGTGAACTTCCCGGCGGACGGCCCCG
TCATGAAGAAGATGACGGACAACTGGGAGCCGTCCTGCGAGAAGATCATCCCGGTGCCCAAGCAGGGCATCCTGAAGGGCGACGTCAGCATGTACCT

CCTGCTCAAGGACGGCGGCAGGCTCAGGTGCCAGTTCGACACGGTGTACAAGGCGAAGTCCGTCCCGAGGAAGATGCCCGACTGGCACTTCATCCA
GCACAAGCTCACCCGCGAGGACAGGAGCGACGCCAAGAACCAGAAGTGGCACCTGACCGAGCACGCTATCGCCTCCGGCAGCGCGCTCCCCTGAGC
TCAAAAAAAAAAAAAAAAAAAAAAAAAAAAAAAAAAAAAAAAAAAAAAAAAAGAATTGGTACC**GTTCACTGCCGTATAGGCAG**CGGGATGGTGGT
GCAGTGGGGTTTTAGAGCTAGAAATAGCAAGTTAAAATAAGGCTAGTCCGTTATCAACTTGAAAAAGTGGCACCGAGTCGGTGCTAGAACCTGTCT
TCTAACTGCACCACCATGGCCGGCATGGTCCCAGCCTCCTCGCTGGCGCCGGCTGGGCAACATGCTTCGGCATGGCGAATGGGACGTTCACTGCCG
TATAGGCAGCCTTGAAAGCCCCACCACTAGTTTTAGAGCTAGAAATAGCAAGTTAAAATAAGGCTAGTCCGTTATCAACTTGAAAAAGTGGCACCG
AGTCGGTGCTGCCTATGATACCTATTGGTGGGGCTTTCGGCCGGCATGGTCCCAGCCTCCTCGCTGGCGCCGGCTGGGCAACATGCTTCGGCATGGC
GAATGGGACGTTCACTGCCGTATAGGCAGCCTAGGGATATCTCCGGGGCTAATTGAATATGAAGATGAAGATGAAATATTTGGTGTGTCAAATAAAAAG
CTGGTGTGCTTAAGTTTGTGTTTTTTTCTTGGCTTGTTGTGTTATGAATTTGTGGCTTTTTCTAATATTAAATGAATGTAAGATCTCATTATAATGAATAAAC
AAATGTTTCTATAATCCATTGTGAATGTTTTGTTGGATCTCTTCTGCAGCATATAACTACTGTATGTGCTATGGTATGGACTATGGAATATGATTAAAGATAA
G

Note: the underlined part comes from the synthetic fragment.

The two pegRNA cassettes in pL2L1-WS-pegR and pZ1WS

35S-CmYLCV-U6-tGly-T1W-sgR-rtT-PBS-HDV-tMet-T1S-sgR-rtT-PBS-HDV-polyT-HSPt

ATGGAGTCAAAGATTCAAATAGAGGACCTAACAGAACTCGCCGTAAAGACTGGCGAACAGTTCATACAGAGTCTCTTACGACTCAATGACAAGAAGAA
AATCTTCGTCAACATGGTGGAGCACGACACACTTGTCTACTCCAAAAATATCAAAGATACAGTCTCAGAAGACCAAAGGGCAATTGAGACTTTTCAACA
AAGGGTAATATCCGGAAACCTCCTCGGATTCCATTGCCCAGCTATCTGTCACTTTATTGTGAAGATAGTGGAAAAGGAAGGTGGCTCCTACAAATGCCA
TCATTGCGATAAAGGAAAGGCCATCGTTGAAGATGCCTCTGCCGACAGTGGTCCCAAAGATGGACCCCCACCCACGAGGAGCATCGTGGAAAAAGAA
GACGTTCCAACCACGTCTTCAAAGCAAGTGGATTGATGTGATTGGCAGACATACTGTCCCACAAATGAAGATGGAATCTGTAAAAGAAAACGCGTGAA
ATAATGCGTCTGACAAAGGTTAGGTCGGCTGCCTTTAATCAATACCAAAGTGGTCCCTACCACGATGGAAAAACTGTGCAGTCGGTTTGGCTTTTTCTG
ACGAACAAATAAGATTCGTGGCCGACAGGTGGGGGTCCACCATGTGAAGGCATCTTCAGACTCCAATAATGGAGCAATGACGTAAGGGCTTACGAAA
TAAGTAAGGGTAGTTTGGGAAATGTCCACTCACCCGTCAGTCTATAAATACTTAGCCCCTCCCTCATTGTTAAGGGAGCAAAATCTCAGAGAGATAGTCC
TAGAGAGAGAAAGAGAGCAAGTAGCCTAGAAGTAGTCAAGGCGGCGAAGTATTCAGGCACGTGGCCAGGAAGAAGAAAAGCCAAGACGACGAAA
ACAGGTAAGAGCTAAGCATCTAGAAAGTTGAAAACAATCTTCAAAAGTCCCACATCGCTTAGATAAGAAAACGAAGCTGAGTTTATATACAGCTAGAGT
CGAAGTAGTGATTGAACAAAGCACCAGTGGTCTAGTGGTAGAATAGTACCCTGCCACGGTACAGACCCGGGTTCGATTCCCGGCTGGTGCACGGGAT
GGTGGTGCAGTGGGGTTTTAGAGCTAGAAATAGCAAGTTAAAATAAGGCTAGTCCGTTATCAACTTGAAAAAGTGGCACCGAGTCGGTGCTAGAA
CCTGTCTTCTAACTGCACCACCATGGCCGGCATGGTCCCAGCCTCCTCGCTGGCGCCGGCTGGGCAACATGCTTCGGCATGGCGAATGGGACAACA
ACAAATCAGAGTGGCGCAGCGGAAGCGTGGTGGGCCCATAACCCACAGGTCCCAGGATCGAAACCTGGCTCTGATACCTTGAAAGCCCCACCACT
AGTTTTAGAGCTAGAAATAGCAAGTTAAAATAAGGCTAGTCCGTTATCAACTTGAAAAAGTGGCACCGAGTCGGTGCTGCCTATGATACCTATTGGT
GGGGCTTTCGGCCGGCATGGTCCCAGCCTCCTCGCTGGCGCCGGCTGGGCAACATGCTTCGGCATGGCGAATGGGACTTTTTTTTGATATCTCCGGGG
CTAATTGAATATGAAGATGAAGATGAAATATTTGGTGTGTCAAATAAAAAGCTGGTGTGCTTAAGTTTGTGTTTTTTTCTTGGCTTGTTGTGTTATGAATT
TGTGGCTTTTTCTAATATTAAATGAATGTAAGATCTCATTATAATGAATAAACAAATGTTTCTATAATCCATTGTGAATGTTTTGTTGGATCTCTTCTGCAGC
ATATAACTACTGTATGTGCTATGGTATGGACTATGGAATATGATTAAAGATAAG

Note: the underlined part comes from the synthetic fragment.

The two sgRNA cassettes in pR1R4-WS-sgR, pZ1WS, and pZ1WS-Csy4

OsU3p-T2W-sgR-OsU3t-TaU3p-T2S-sgR-TaU3t

AGTAATTCATCCAGGTCACCAAGTTCTAGGATTTTCAGAACTGCAACTTATTTTATCAAGGAATCTTTAAACATACGAACAGATCACTTAAAGTTCTTCTG
AAGCAACTTAAAGTTATCAGGCTTGCATGGATCTTGGAGGAATCAGATGTGCAGTCAGGGACCATAGCACAAGACAGGCGTCTTCTACTGGTGCTACC
AGCAAATGCTGGAAGCCGGGAACACTGGGTACGTTGGAAACCACGTGATGTGAAGAAGTAAGATAAACTGTAGGAGAAAAGCATTTCGTAGTGGGC
CATGAAGCCTTTCAGGACATGTATTGCAGTATGGGCCGGCCCATTACGCAATTGGACGACAACAAAGTCTAGTATTAGTACCACCTCGGCTATCCACATA
GATCAAAGCTGATTTAAAAGAGTTGTGCAGATGATCCGTGGCGTAACTGCACCACCATCCCGGTTTTAGAGCTAGAAATAGCAAGTTAAAATAAGGC
TAGTCCGTTATCAACTTGAAAAAGTGGCACCGAGTCGGTGCTTTTTTTTTTCGTTTTGCATTGAGTTTTCTCCGTCGCATGTTTGCAGCATGAATCCAA
ACCACACGGAGTTCAAATTCCCACAGATTAAGGCTCGTCCGTCGCACAAGGTAATGTGTGAATATTATATCTGTCGTGCAAAATTGCCTGGCCTGCACAA
TTGCTGTTATAGTTGGCGGCAGGGAGAGTTTTAACATTGACTAGCGTGCTGATAATTTGTGAGAAATAATAATTGACAAGTAGATACTGACATTTGAGA
AGAGCTTCTGAACTGTTATTAGTAACAAAAATGGAAAGCTGATGCACGGAAAAAGGAAAGAAAAAGCCATACTTTTTTTTAGGTAGGAAAAGAAAAA
GCCATACGAGACTGATGTCTCTCAGATGGGCCGGGATCTGTCTATCTAGCAGGCAGCAGCCCACCAACCTCACGGGCCAGCAATTACGAGTCCTTCTAA
AAGCTCCCGCCGAGGGGCGCTGGCGCTGCTGTGCAGCAGCACGTCTAACATTAGTCCCACCTCGCCAGTTTACAGGGAGCAGAACCAGCTTATAAGC
GGAGGCGCGGCACCAAGAAGCGATATGATCCTGGATGGTGAGTTTTAGAGCTAGAAATAGCAAGTTAAAATAAGGCTAGTCCGTTATCAACTTGAAA
AAGTGGCACCGAGTCGGTGCTTTTTTTTTTGTCCTTCTGTTTTTTTAGTCAGTCTCTTTTTTCAGAAGTACAACATCTT

Note: the underlined part comes from the synthetic fragment.

The pegRNA cassettes in pU3-ALS-S1 and pU3-GAPDH

OsU3p-tGly-Spacer-sgR-rtT-PBS-HDV-TaU3t

AGTAATTCATCCAGGTCACCAAGTTCTAGGATTTTCAGAACTGCAACTTATTTTATCAAGGAATCTTTAAACATACGAACAGATCACTTAAAGTTCTTCTG
AAGCAACTTAAAGTTATCAGGCTTGCATGGATCTTGGAGGAATCAGATGTGCAGTCAGGGACCATAGCACAAGACAGGCGTCTTCTACTGGTGCTACC
AGCAAATGCTGGAAGCCGGGAACACTGGGTACGTTGGAAACCACGTGATGTGAAGAAGTAAGATAAACTGTAGGAGAAAAGCATTTCGTAGTGGGC
CATGAAGCCTTTCAGGACATGTATTGCAGTATGGGCCGGCCCATTACGCAATTGGACGACAACAAAGTCTAGTATTAGTACCACCTCGGCTATCCACATA
GATCAAAGCTGATTTAAAAGAGTTGTGCAGATGATCCGTGGCAACAAAGCACCAGTGGTCTAGTGGTAGAATAGTACCCTGCCACGGTACAGACCCGG
GTTCGATTCCCGGCTGGTGCANNNNNNNNNNNNNNNNNNNNGTTTTAGAGCTAGAAATAGCAAGTTAAAATAAGGCTAGTCCGTTATCAACTTG
AAAAAGTGGCACCGAGTCGGTGCNNNNNNNNNNNNNNNNNNNNNNNNNNNNNGGCCGGCATGGTCCCAGCCTCCTCGCTGGCGCCGGCTGG
GCAACATGCTTCGGCATGGCGAATGGGACTTTTTTTTTTGTCCTTCTGTTTTTTTAGTCAGTCTCTTTTTTCAGAAGTACAACATCTT
Note: the underlined part comes from the synthetic fragments.

The pegRNA cassettes in p35C-ALS-S1 and p35C-GAPDH

35S-CmYLCV-U6-tGly-Spacer-sgR-rtT-PBS-HDV-polyT-HSPt
ATGGAGTCAAAGATTCAAATAGAGGACCTAACAGAACTCGCCGTAAAGACTGGCGAACAGTTCATACAGAGTCTCTTACGACTCAATGACAAGAAGAA
AATCTTCGTCAACATGGTGGAGCACGACACACTTGTCTACTCCAAAAATATCAAAGATACAGTCTCAGAAGACCAAAGGGCAATTGAGACTTTTCAACA
AAGGGTAATATCCGGAAACCTCCTCGGATTCCATTGCCCAGCTATCTGTCACTTTATTGTGAAGATAGTGGAAAAGGAAGGTGGCTCCTACAAATGCCA
TCATTGCGATAAAGGAAAGGCCATCGTTGAAGATGCCTCTGCCGACAGTGGTCCCAAAGATGGACCCCCACCCACGAGGAGCATCGTGGAAAAAGAA
GACGTTCCAACCACGTCTTCAAAGCAAGTGGATTGATGTGATTGGCAGACATACTGTCCCACAAATGAAGATGGAATCTGTAAAAGAAAACGCGTGAA
ATAATGCGTCTGACAAAGGTTAGGTCGGCTGCCTTTAATCAATACCAAAGTGGTCCCTACCACGATGGAAAAACTGTGCAGTCGGTTTGGCTTTTTCTG
ACGAACAAATAAGATTCGTGGCCGACAGGTGGGGGTCCACCATGTGAAGGCATCTTCAGACTCCAATAATGGAGCAATGACGTAAGGGCTTACGAAA
TAAGTAAGGGTAGTTTGGGAAATGTCCACTCACCCGTCAGTCTATAAATACTTAGCCCCTCCCTCATTGTTAAGGGAGCAAAATCTCAGAGAGATAGTCC
TAGAGAGAGAAAGAGAGCAAGTAGCCTAGAAGTAGTCAAGGCGGCGAAGTATTCAGGCACGTGGCCAGGAAGAAGAAAAGCCAAGACGACGAAA
ACAGGTAAGAGCTAAGCATCTAGAAAGTTGAAAACAATCTTCAAAAGTCCCACATCGCTTAGATAAGAAAACGAAGCTGAGTTTATATACAGCTAGAGT
CGAAGTAGTGATTGAACAAAGCACCAGTGGTCTAGTGGTAGAATAGTACCCTGCCACGGTACAGACCCGGGTTCGATTCCCGGCTGGTGCANNNNNN
NNNNNNNNNNNNNNGTTTTAGAGCTAGAAATAGCAAGTTAAAATAAGGCTAGTCCGTTATCAACTTGAAAAAGTGGCACCGAGTCGGTGCNNN
NNNNNNNNNNNNNNNNNNNNNNNNNNGGCCGGCATGGTCCCAGCCTCCTCGCTGGCGCCGGCTGGGCAACATGCTTCGGCATGGCGAATGGG
ACTTTTTTTTGATATCTCCGGGGCTAATTGAATATGAAGATGAAGATGAAATATTTGGTGTGTCAAATAAAAAGCTGGTGTGCTTAAGTTTGTGTTTTTTT
CTTGGCTTGTTGTGTTATGAATTTGTGGCTTTTTCTAATATTAAATGAATGTAAGATCTCATTATAATGAATAAACAAATGTTTCTATAATCCATTGTGAATG
TTTTGTTGGATCTCTTCTGCAGCATATAACTACTGTATGTGCTATGGTATGGACTATGGAATATGATTAAAGATAAG
Note: the underlined part comes from the synthetic fragment.

The pegRNA cassettes in p2xU3-ALS-WS, pG3R23-OsWS, pR1R4-OsWS, and pL4L3-OsWS

OsU3p-tGly-Spacer-sgR-rtT-PBS-HDV-OsU3t-TaU3p-tMet-Spacer-sgR-rtT-PBS-HDV-TaU3t
AGTAATTCATCCAGGTCACCAAGTTCTAGGATTTTCAGAACTGCAACTTATTTTATCAAGGAATCTTTAAACATACGAACAGATCACTTAAAGTTCTTCTG
AAGCAACTTAAAGTTATCAGGCTTGCATGGATCTTGGAGGAATCAGATGTGCAGTCAGGGACCATAGCACAAGACAGGCGTCTTCTACTGGTGCTACC
AGCAAATGCTGGAAGCCGGGAACACTGGGTACGTTGGAAACCACGTGATGTGAAGAAGTAAGATAAACTGTAGGAGAAAAGCATTTCGTAGTGGGC
CATGAAGCCTTTCAGGACATGTATTGCAGTATGGGCCGGCCCATTACGCAATTGGACGACAACAAAGTCTAGTATTAGTACCACCTCGGCTATCCACATA
GATCAAAGCTGATTTAAAAGAGTTGTGCAGATGATCCGTGGCAACAAAGCACCAGTGGTCTAGTGGTAGAATAGTACCCTGCCACGGTACAGACCCGG
GTTCGATTCCCGGCTGGTGCAGGGTATGGTGGTGCAATGGGGTTTTAGAGCTAGAAATAGCAAGTTAAAATAAGGCTAGTCCGTTATCAACTTGAA
AAAGTGGCACCGAGTCGGTGCAAACCTATCTTCTAATTGCACCACCATGGCCGGCATGGTCCCAGCCTCCTCGCTGGCGCCGGCTGGGCAACATGC
TTCGGCATGGCGAATGGGACTTTTTTTTTTCGTTTTGCATTGAGTTTTCTCCGTCGCATGTTTGCAGTTTTATTTTCCGTTTTGCATTGAAATTTCTCCG
TCTCATGTTTGC**AG**CGTGTTCAACATGAATCCAAACCACACGGAGTTCAAATTCCCACAGATTAAGGCTCGTCCGTCGCACAAGGTAATGTGTGAAT
ATTATATCTGTCGTGCAAAATTGCCTGGCCTGCACAATTGCTGTTATAGTTGGCGGCAGGGAGAGTTTTAACATTGACTAGCGTGCTGATAATTTGT
GAGAAATAATAATTGACAAGTAGATACTGACATTTGAGAAGAGCTTCTGAACTGTTATTAGTAACAAAAATGGAAAGCTGATGCACGGAAAAAGG
AAAGAAAAAGCCATACTTTTTTTTAGGTAGGAAAAGAAAAAGCCATACGAGACTGATGTCTCTCAGATGGGCCGGGATCTGTCTATCTAGCAGGCA
GCAGCCCACCAACCTCACGGGCCAGCAATTACGAGTCCTTCTAAAAGCTCCCGCCGAGGGGCGCTGGCGCTGCTGTGCAGCAGCACGTCTAACATT
AGTCCCACCTCGCCAGTTTACAGGGAGCAGAACCAGCTTATAAGCGGAGGCGCGGCACCAAGAAGCGAACAACAAAACAACAAATCAGAGTGGC
GCAGCGGAAGCGTGGTGGGCCCATAACCCACAGGTCCCAGGATCGAAACCTGGCTCTGATACCTTGAATGCGCCCCCACTTGTTTTAGAGCTAGAA
ATAGCAAGTTAAAATAAGGCTAGTCCGTTATCAACTTGAAAAAGTGGCACCGAGTCGGTGCTGCCTATGATACCAATTGGGGGCGCATTCGGCCGG
CATGGTCCCAGCCTCCTCGCTGGCGCCGGCTGGGCAACATGCTTCGGCATGGCGAATGGGACTTTTTTTTTTGTCCTTCTGTTTTTTTAGTCAGTCTCT
TTTTTCAGAAGTACAACATCTT
Note: the underlined part comes from the synthetic fragments.

The pegRNA cassettes in p35C-ALS-WS and pL2L1-OsWS

35S-CmYLCV-U6-tGly-Spacer-sgR-rtT-PBS-HDV-tMet-Spacer-sgR-rtT-PBS-HDV-polyT-HSPt
ATGGAGTCAAAGATTCAAATAGAGGACCTAACAGAACTCGCCGTAAAGACTGGCGAACAGTTCATACAGAGTCTCTTACGACTCAATGACAAGAAGAA
AATCTTCGTCAACATGGTGGAGCACGACACACTTGTCTACTCCAAAAATATCAAAGATACAGTCTCAGAAGACCAAAGGGCAATTGAGACTTTTCAACA

AAGGGTAATATCCGGAAACCTCCTCGGATTCCATTGCCCAGCTATCTGTCACTTTATTGTGAAGATAGTGGAAAAGGAAGGTGGCTCCTACAAATGCCA
TCATTGCGATAAAGGAAAGGCCATCGTTGAAGATGCCTCTGCCGACAGTGGTCCCAAAGATGGACCCCCACCCACGAGGAGCATCGTGGAAAAAGAA
GACGTTCCAACCACGTCTTCAAAGCAAGTGGATTGATGTGATTGGCAGACATACTGTCCCACAAATGAAGATGGAATCTGTAAAAGAAAACGCGTGAA
ATAATGCGTCTGACAAAGGTTAGGTCGGCTGCCTTTAATCAATACCAAAGTGGTCCCTACCACGATGGAAAAACTGTGCAGTCGGTTTGGCTTTTTCTG
ACGAACAAATAAGATTCGTGGCCGACAGGTGGGGGTCCACCATGTGAAGGCATCTTCAGACTCCAATAATGGAGCAATGACGTAAGGGCTTACGAAA
TAAGTAAGGGTAGTTTGGGAAATGTCCACTCACCCGTCAGTCTATAAATACTTAGCCCCTCCCTCATTGTTAAGGGAGCAAAATCTCAGAGAGATAGTCC
TAGAGAGAGAAAGAGAGCAAGTAGCCTAGAAGTAGTCAAGGCGGCGAAGTATTCAGGCACGTGGCCAGGAAGAAGAAAAGCCAAGACGACGAAA
ACAGGTAAGAGCTAAGCATCTAGAAAGTTGAAAACAATCTTCAAAAGTCCCACATCGCTTAGATAAGAAAACGAAGCTGAGTTTATATACAGCTAGAGT
CGAAGTAGTGATTGAACAAAGCACCAGTGGTCTAGTGGTAGAATAGTACCCTGCCACGGTACAGACCCGGGTTCGATTCCCGGCTGGTGCAGGGTAT
GGTGGTGCAATGGGGTTTTAGAGCTAGAAATAGCAAGTTAAAATAAGGCTAGTCCGTTATCAACTTGAAAAAGTGGCACCGAGTCGGTGCAAACC
TATCTTCTAATTGCACCACCATGGCCGGCATGGTCCCAGCCTCCTCGCTGGCGCCGGCTGGGCAACATGCTTCGGCATGGCGAATGGGACAACAAC
AAATCAGAGTGGCGCAGCGGAAGCGTGGTGGGCCCATAACCCACAGGTCCCAGGATCGAAACCTGGCTCTGATACCTTGAATGCGCCCCCACTTGT
TTTAGAGCTAGAAATAGCAAGTTAAAATAAGGCTAGTCCGTTATCAACTTGAAAAAGTGGCACCGAGTCGGTGCTGCCTATGATACCAATTGGGGG
CGCATTCGGCCGGCATGGTCCCAGCCTCCTCGCTGGCGCCGGCTGGGCAACATGCTTCGGCATGGCGAATGGGACTTTTTTTTGATATCTCCGGGGCT
AATTGAATATGAAGATGAAGATGAAATATTTGGTGTGTCAAATAAAAAGCTGGTGTGCTTAAGTTTGTGTTTTTTTCTTGGCTTGTTGTGTTATGAATTTG
TGGCTTTTTCTAATATTAAATGAATGTAAGATCTCATTATAATGAATAAACAAATGTTTCTATAATCCATTGTGAATGTTTTGTTGGATCTCTTCTGCAGCATA
TAACTACTGTATGTGCTATGGTATGGACTATGGAATATGATTAAAGATAAG

Note: the underlined part comes from the synthetic fragment.
