# Supplementary material for: Prime editing efficiently generates W542L and S621I double mutations in two ALS genes in maize
Source: Genome Biol. 2020 Oct 6;21:257. doi: 10.1186/s13059-020-02170-5 (PMC7541250; doi:10.1186/s13059-020-02170-5)
Supplement: Supplementary file 2 — Additional file 2. Review history. [file 13059_2020_2170_MOESM2_ESM.docx]

Review History

**First round of review**

**Reviewer 1**

**Are you able to assess all statistics in the manuscript, including the appropriateness of statistical tests used?** Yes, and I have assessed the statistics in my report.

**Comments to author:**

In this manuscript, Jiang et al established the prime editing system in maize. This is the first report on the application of prime editing in maize, an important crop worldwide. In contrast to the low prime editing efficiency reported in rice, Jiang et al achieved higher prime editing efficiency in maize by enhancing the pegRNA expression. I think their results are inspiring and provide a direction for further optimization of prime editors in other organisms. But some concerns need to be addressed before publication in Genome Biology.
1.     The experiments were not well-designed in this study. It lacks a standard control for the pZ1WS editor to support the conclusion that enhancing the pegRNA expression can achieve much higher prime editing efficiency in maize. The pZ1WS editor could not directly compare with pZ1PE3 and pZ1PE3b editors because they used different target sites. An appropriate control for the pZ1WS editor at the W542L/S621I target sites was a prime editor that did not contain the two promoter systems driving the pegRNAs expression.
2.     Did the authors try to edit the P165S site by using the pZ1WS editor? If the pZ1WS editor can edit the P165S site, it also supports the main conclusion of this study.
3.     The expression level of a modified sgRNA scaffold (esgRNA) is higher than that of normal sgRNA scaffold. A previous study in rice (Xu et al., 2020) showed that the pegRNA with an esgRNA scaffold can improve the prime editing efficiency at most target sites except one, suggesting that enhancing pegRNA expression can increase prime editing efficiency. However, the pZ1WS editor only edited one gene in this study and its higher editing efficiency in maize needs to be verified by more targets. The authors can test more targets in protoplasts.
4.     Did the author check the expression level of pegRNA when it was driven by different promoters? Could the author explain the reason to use the double polymerase II (35S enhancer-CmYLCV) and III (shortened U6-26) promoters to drive pegRNA expression? The two RNA polymerases will competitively bind to this CmYLCV-U6 double-promoter that may affect transcription. Was the double promoters much better than single one?
5.     The OsU3 and TaU3 promoters are demonstrated to be strong enough to drive expression of sgRNA that can support the CRISPR-Cas9 mediated knockout and base editing in maize. It seems like that pegRNA expression driven by OsU3 alone can't support prime editing in maize. Why does the prime editing need the extremely high expression level of pegRNA to achieve efficient editing in maize? The authors need briefly discuss this in the main text.
6.     The mechnism underlying the high editing efficiency of pZ1WS editor is unknown. The authors think that the higher expression level of pegRNA is the main reason, but an alternative explanation is that the tRNA and cHDV ribozyme may help to stabilize or protect the 3'-terminal of pegRNA and thus improve the prime editing efficiency.

**Reviewer 2**

**Are you able to assess all statistics in the manuscript, including the appropriateness of statistical tests used?** Yes, and I have assessed the statistics in my report.

**Comments to author:**

In this article, Jiang, et al demonstrate efficient prime editing in maize and describe optimizations to pegRNA expression vectors that enable enhanced editing efficiency. The authors tested previously reported pegRNA expression systems based on U3 promoters, as well as designs that use Csy4/HDV ribozyme processing or tRNA/HDV ribozyme processing. The authors found that, while the previously reported expression systems and Csy4-based systems did not produce efficient prime editing at the tested genomic sites, the tRNA/HDV ribozyme expression systems supported efficient prime edits. This enabled the simultaneous installation of two distinct point mutations into ZmALS1 and ZmALS2 genes. This manuscript reports important developments for the application of prime editing to maize, which should be broadly useful to the plant genome editing community.

Major Comments:

1). Prior studies have found that prime editing efficiency can vary based on the target locus, intended edit, and pegRNA design (PBS and RT template sequences). While the new pegRNA expression strategy (vector pZ1WS) led to relatively higher editing efficiency, it appears that it was not directly compared to previously reported expression systems to perform the same edit with identical pegRNA designs. A direct comparison would better support the claim that the enhanced editing observed using pZ1WS vs. pZ1PE3 was a result of an improved pegRNA expression system and not merely due to a more amenable target sequence and edit.

2). The authors should consider using their improved pegRNA expression system to test a broader set of pegRNA sequence designs for unsuccessful edits such as the P165S edit.

3). The authors report that incorporation of reverse transcribed scaffold sequence into genomic DNA was observed in some editing products. They should clarify the exact nature of these edits, i.e. was scaffold-derived sequence observed within indels, or were they found as substitutions at corresponding positions in the DNA. In addition, the authors should comment on the presence of (or their ability to detect) other byproducts, such as indels.      

Minor Comments:

1). The figures are low resolution and should be enhanced.

2). In the last paragraph of the results section, the authors propose a strategy for reducing scaffold-derived byproducts by designing pegRNAs so that the terminal 1 to 3 nucleotides of the scaffold sequence match the target genomic site. However, data supporting this strategy was not presented. The authors should include data that supports this claim, or the statement should be moved from the results section to the discussion/conclusion section and language should be revised to make it clear that this is a potential strategy for reducing scaffold incorporation byproducts.

**Reviewer 3**

**Are you able to assess all statistics in the manuscript, including the appropriateness of statistical tests used?** Yes, and I have assessed the statistics in my report.

**Comments to author:**

Prime editing, as a breakthrough in genome editing technology, is promising in realizing predefined, precise, and flexible editing both in animals and plants. However,  low editing efficiency is by bar the bottleneck in plant prime editing. In this study, the authors achieved high prime editing efficiency in maize via enhancing pegRNA expression. Though the gene editing toolkit generated by Jiang et al seems promising, the manuscripts falls short of some major shortcomings listed below. A re-evaluation is necessary and the authors should submit a majorly revised version by addressing following comments.

Major comment:
(1) Experimental design of the manuscript is not very rigorous. For example, the P165S editing event was performed in both pZ1PE3 and pZ1PE3b vector, while unsuccessful desired edits were found in the transformation events. However, why not carry out the P165S editing event to compare the editing efficiency in pZ1WS vector. Moreover, the desired edits were just found in the transgenic lines transformed with pZ1WS, while not in the transgenic lines both transformed with pZ1PE3 and pZ1PE3b. Therefore, whether to generate the desired edits only need to use two promoter systems to drive pegRNA expression, together with the use of tRNA and ribozyme processing systems, without the need for additional pegRNA expression cassettes. Additionally, 16 transgenic lines are too few compared to the control.
(2) PE system produces various editing efficiencies at different target sites in previous studies. And this phenomenon is also reflected in Figure 2C. In my opinion, it is meaningless to perform two repeatedly transformation for plasmid pZ1WS targeting W542L and S621I in maize. Instead, it is proper to edit some other genome loci to further verify the efficiency of this system.
(3) Among 129 plants detected, Li et al (2020, Mol Plant) achieved 15 individual homozygous plants carrying the precise edits. However, this is not mentioned in background. Instead, it mentions another example of inefficient editing. So in my opinion, this background is not comprehensive.

Minor comment:
(1) In the Conclusion section, does the number of transgenic lines '62' mean the sum of two repeatedly transformation? If so, it is not reasonable because of the two independent experiments.
(2) "For the W542L edits, the sgRNA nicking non-edited strand fully matches the ZmALS1 target" , the description is incorrect. Nicking sgRNAs of PE3b with spacers match the edited strand, but not the original allele.
(3) "In addition, we achieved two additional lines harboring the S621I homozygous mutations in the two ALS genes", the Sanger sequencing diagram of two S621I homozygous mutations lines should be provided.
(4) What does "double-strand even heteroduplex DNA repair" mean? Because no specific explanation is given in this article.
(5) The Figures looks confusing, further modification and improvement need to be done.

**Authors Response**

## Response #E1.

Thanks a lot. The three reviewers’ main concerns involve our experimental design and science rigorousness of our conclusions. We completely understand these concerns, have given detailed explanation in our response, and revised our manuscript accordingly.

Since maize transformation requires a long period (> 3 months) and the research is highly competitive, the maize transformation experiments will severely delay the publication and thus lead to lose the novelty. Testing in maize protoplasts seem a good idea, however, the results are not always stable, especially when vectors have a large size (the size of pZ1WS is 24.7-kb). Therefore, we turned to testing in rice protoplasts and cooperated with Dr. Caixia Gao for the experiments. The results indicated: (1) the strategy based on CaMV35S-CmYLCV-U6 composite promoter significantly improved the prime-editing efficiencies for three of four pegRNAs; (2) the strategy based on redoubling the number of pegRNA cassettes had no contributions to improving prime-editing efficiency (Additional file 1: Fig. S3 and Table S4).

# Response to Reviewers

# Reviewer #1:

In this manuscript, Jiang et al established the prime editing system in maize. This is the first report on the application of prime editing in maize, an important crop worldwide. In contrast to the low prime editing efficiency reported in rice, Jiang et al achieved higher prime editing efficiency in maize by enhancing the pegRNA expression. I think their results are inspiring and provide a direction for further optimization of prime editors in other organisms. But some concerns need to be addressed before publication in Genome Biology.

1. The experiments were not well-designed in this study. It lacks a standard control for the pZ1WS editor to support the conclusion that enhancing the pegRNA expression can achieve much higher prime editing efficiency in maize. The pZ1WS editor could not directly compare with pZ1PE3 and pZ1PE3b editors because they used different target sites. An appropriate control for the pZ1WS editor at the W542L/S621I target sites was a prime editor that did not contain the two promoter systems driving the pegRNAs expression.

## Response #1-1.

Thanks a lot. Yes, pZ1PE3 and pZ1PE3b should not be used as controls for pZ1WS. Actually, we constructed two optimized prime editors based on Csy4 system, p4xZ1PE3-Csy4 and p4xZ1PE3b-Csy4, and pZ1PE3 and pZ1PE3b were the controls of these two Csy4-based editors harboring redoubled pegRNA and sgRNA expression cassettes. However, the transformations of Csy4 vectors including these two were unexpectedly unsuccessful, which we attribute to the toxicity of Csy4 protein in maize. To clarify the doubt to the experimental design, we described these two plasmids in the revised main text. We designed the pZ1WS-Csy4 and pZ1WS editors for an attempt to generate herbicide-resistance maize lines rather than for comparison of editing efficiencies. This is the reason why these two editors lacked controls harboring single pegRNA cassettes. The pZ1WS vector was constructed as a control of pZ1WS-Csy4 for comparing the non-Csy4 system with the Csy4 system. We should have constructed more editors including control vectors of pZ1WS especially after we achieved high editing efficiency with pZ1WS. However, maize transformation requires a long period (> 3 months) and too long delay of the publication will lead to lose the novelty. Testing in maize protoplasts seems a good idea, however, the maize protoplast transfection results are not always stable, especially when vectors have a large size (the size of pZ1WS is 24.7-kb). Therefore, we turned to testing in rice protoplasts and cooperated with Dr. Caixia Gao for the experiments. The results indicated: (1) the strategy based on CaMV35S-CmYLCV-U6 composite promoter significantly improved the prime-editing efficiencies for three of four pegRNAs; (2) the strategy based on redoubling the number of pegRNA cassettes had no contributions to improving prime-editing efficiency (Additional file 1: Fig. S3 and Table S4).

2. Did the authors try to edit the P165S site by using the pZ1WS editor? If the pZ1WS editor can edit the P165S site, it also supports the main conclusion of this study.

## Response #1-2.

Thanks a lot. Actually, we constructed two optimized prime editors (p4xZ1PE3-Csy4 and p4xZ1PE3b-Csy4) harboring the P165S site with the structure similar to pZ1WS-Csy4, but unfortunately, the maize transformations were unsuccessful.

We should have constructed more editors including controls. However, maize transformation requires a long period (> 3 months) and too long delay of the publication will lead to lose the novelty. Testing in maize protoplasts seems a good idea, however, the maize protoplast transfection results are not always stable, especially when vectors have a large size (the size of pZ1WS is 24.7-kb). Therefore, we turned to testing in rice protoplasts and cooperated with Dr. Caixia Gao for the experiments. The results indicated: (1) the strategy based on CaMV35S-CmYLCV-U6 composite promoter significantly improved the prime-editing efficiencies for three of four pegRNAs; (2) the strategy based on redoubling the number of pegRNA cassettes had no contributions to improving prime-editing efficiency (Additional file 1: Fig. S3 and Table S4).

3. The expression level of a modified sgRNA scaffold (esgRNA) is higher than that of normal sgRNA scaffold. A previous study in rice (Xu et al., 2020) showed that the pegRNA with an esgRNA scaffold can improve the prime editing efficiency at most target sites except one, suggesting that enhancing pegRNA expression can increase prime editing efficiency. However, the pZ1WS editor only edited one gene in this study and its higher editing efficiency in maize needs to be verified by more targets. The authors can test more targets in protoplasts.

## Response #1-3.

Thanks a lot. We are not convinced of the Yang Lab's results that the mutant sgRNA scaffold displayed better than the original one. In comparison with the original version PE-P1, the improved version PE-P2 was improved from three aspects: (1) the promoter of PE2 was changed from OsUbi to ZmUbi; (2) selection marker gene Hyg was fused to PE2 in PE-P2 for enriching purpose whereas it was expressed independently in PE-P1. (3) the sgRNA scaffold was changed from the original one to a mutant one. Obviously, the results can’t conclude that the mutant sgRNA scaffold is the only or main reason for the improved editing efficiency. We tested two nonallelic targets of two ALS genes, which represents a more challenging task for prime editing. Of course, the more targets we tested, the higher quality of the results we could achieved.

Testing in maize protoplasts seems a good idea, however, the results are not always stable, especially when vectors have a large size (the size of pZ1WS is 24.7-kb). Therefore, we turned to testing in rice protoplasts and cooperated with Dr. Caixia Gao for the experiments. The results indicated: (1) the strategy based on CaMV35S-CmYLCV-U6 composite promoter significantly improved the prime-editing efficiencies for three of four pegRNAs; (2) the strategy based on redoubling the number of pegRNA cassettes had no contributions to improving prime-editing efficiency (Additional file 1: Fig. S3 and Table S4).

4. Did the author check the expression level of pegRNA when it was driven by different promoters? Could the author explain the reason to use the double polymerase II (35S enhancer-CmYLCV) and III (shortened U6-26) promoters to drive pegRNA expression? The two RNA polymerases will competitively bind to this CmYLCV-U6 double-promoter that may affect transcription. Was the double promoters much better than single one?

## Response #1-4.

Thanks a lot. We have not checked the expression level of pegRNA. We focused on the applications of PE in plants and improvement of the prime editing efficiency was our first-priority. Obviously, the reason why we used the Pol2 and Pol3 composite promoter is to attempt to enhance the pegRNA expression. Yes, it is necessary to compare the expression strength of the composite promoter with the single Pol2 promoter in the future. In addition, in the revised manuscript, we provided testing results in the rice protoplasts and the results indicated the strategy based on CaMV35S-CmYLCV-U6 composite promoter significantly improved the prime-editing efficiencies for three of four pegRNAs (Additional file 1: Fig. S3 and Table S4).

5. The OsU3 and TaU3 promoters are demonstrated to be strong enough to drive expression of sgRNA that can support the CRISPR-Cas9 mediated knockout and base editing in maize. It seems like that pegRNA expression driven by OsU3 alone can't support prime editing in maize. Why does the prime editing need the extremely high expression level of pegRNA to achieve efficient editing in maize? The authors need briefly discuss this in the main text.

## Response #1-5.

Thanks a lot. We have explained this in the original text: “According to the structure of pegRNAs, a hairpin can potentially form between the PBS and the protospacer of a pegRNA (Fig. 1a), and thus will possibly weaken the pegRNA activity. Based on this possibility, we reasoned that enhancing pegRNA expression will be able to improve editing efficiency”.

6. The mechnism underlying the high editing efficiency of pZ1WS editor is unknown. The authors think that the higher expression level of pegRNA is the main reason, but an alternative explanation is that the tRNA and cHDV ribozyme may help to stabilize or protect the 3'-terminal of pegRNA and thus improve the prime editing efficiency.

## Response #1-6.

Thanks a lot. To figure out the mechanisms, we generated seven rice prime editors based on different strategies for pegRNA expression and tested them in rice protoplasts. The results indicated: (1) the strategy based on CaMV35S-CmYLCV-U6 composite promoter significantly improved the prime-editing efficiencies for three of four pegRNAs; (2) the strategy based on redoubling the number of pegRNA cassettes had no contributions to improving prime-editing efficiency (Additional file 1: Fig. S3 and Table S4).

# Reviewer #2:

In this article, Jiang, et al demonstrate efficient prime editing in maize and describe optimizations to pegRNA expression vectors that enable enhanced editing efficiency. The authors tested previously reported pegRNA expression systems based on U3 promoters, as well as designs that use Csy4/HDV ribozyme processing or tRNA/HDV ribozyme processing. The authors found that, while the previously reported expression systems and Csy4-based systems did not produce efficient prime editing at the tested genomic sites, the tRNA/HDV ribozyme expression systems supported efficient prime edits. This enabled the simultaneous installation of two distinct point mutations into ZmALS1 and ZmALS2 genes. This manuscript reports important developments for the application of prime editing to maize, which should be broadly useful to the plant genome editing community.

Major Comments:

1). Prior studies have found that prime editing efficiency can vary based on the target locus, intended edit, and pegRNA design (PBS and RT template sequences). While the new pegRNA expression strategy (vector pZ1WS) led to relatively higher editing efficiency, it appears that it was not directly compared to previously reported expression systems to perform the same edit with identical pegRNA designs. A direct comparison would better support the claim that the enhanced editing observed using pZ1WS vs. pZ1PE3 was a result of an improved pegRNA expression system and not merely due to a more amenable target sequence and edit.

## Response #2-1.

Thanks a lot. Yes, pZ1PE3 and pZ1PE3b should not be used as controls for pZ1WS. Actually, we constructed two optimized prime editors based on Csy4 system, p4xZ1PE3-Csy4 and p4xZ1PE3b-Csy4, and pZ1PE3 and pZ1PE3b were the controls of these two Csy4-based editors harboring redoubled pegRNA and sgRNA expression cassettes. However, the transformations of Csy4 vectors including these two were unexpectedly unsuccessful, which we attribute to the toxicity of Csy4 protein in maize. To clarify the doubt to the experimental design, we described these two plasmids in the revised main text. We designed the pZ1WS-Csy4 and pZ1WS editors for an attempt to generate herbicide-resistance maize lines rather than for comparison of editing efficiencies. This is the reason why these two editors lacked controls harboring single pegRNA cassettes. The pZ1WS vector was constructed as a control of pZ1WS-Csy4 for comparing the non-Csy4 system with the Csy4 system. We should have constructed more editors including controls especially after we achieved high editing efficiency with pZ1WS. However, maize transformation requires a long period (> 3 months) and too long delay of the publication will lead to lose the novelty. Testing in maize protoplasts seems a good idea, however, the maize protoplast transfection results are not always stable, especially when vectors have a large size (the size of pZ1WS is 24.7-kb). Therefore, we turned to testing in rice protoplasts and cooperated with Dr. Caixia Gao for the experiments. The results indicated: (1) the strategy based on CaMV35S-CmYLCV-U6 composite promoter significantly improved the prime-editing efficiencies for three of four pegRNAs; (2) the strategy based on redoubling the number of pegRNA cassettes had no contributions to improving prime-editing efficiency (Additional file 1: Fig. S3 and Table S4).

2). The authors should consider using their improved pegRNA expression system to test a broader set of pegRNA sequence designs for unsuccessful edits such as the P165S edit.

## Response #2-2.

Thanks a lot. We tested two nonallelic targets of two ALS genes, which represents a more challenging task for prime editing. Of course, the more targets we tested, the higher quality of the results we could achieved. However, maize transformation requires a long period (> 3 months) and too long delay of publication will lead to lose the novelty. Testing in maize protoplasts seems a good idea, however, the maize protoplast transfection results are not always stable, especially when vectors have a large size (the size of pZ1WS is 24.7-kb). Therefore, we turned to testing in rice protoplasts and cooperated with Dr. Caixia Gao for the experiments. The results indicated: (1) the strategy based on CaMV35S-CmYLCV-U6 composite promoter significantly improved the prime-editing efficiencies for three of four pegRNAs; (2) the strategy based on redoubling the number of pegRNA cassettes had no contributions to improving prime-editing efficiency (Additional file 1: Fig. S3 and Table S4).

3). The authors report that incorporation of reverse transcribed scaffold sequence into genomic DNA was observed in some editing products. They should clarify the exact nature of these edits, i.e. was scaffold-derived sequence observed within indels, or were they found as substitutions at corresponding positions in the DNA. In addition, the authors should comment on the presence of (or their ability to detect) other byproducts, such as indels.

## Response #2-3.

Thanks a lot. The pegRNA scaffold-derived byproducts were also described in previous reports by David Liu Lab and Caixia Gao Lab. This type of byproducts can be easily differentiated from indels. Surprisingly, we observed no indel byproducts and we added the statement to the text.

Minor Comments:

1). The figures are low resolution and should be enhanced.

## Response #2-1b.

Thanks a lot. The low resolution is owing to the conversion program and the original figure files do have the required resolution.

2). In the last paragraph of the results section, the authors propose a strategy for reducing scaffold-derived byproducts by designing pegRNAs so that the terminal 1 to 3 nucleotides of the scaffold sequence match the target genomic site. However, data supporting this strategy was not presented. The authors should include data that supports this claim, or the statement should be moved from the results section to the discussion/conclusion section and language should be revised to make it clear that this is a potential strategy for reducing scaffold incorporation byproducts.

## Response #2-2b.

Thanks a lot. The results and discussion sections were combined according to the requirement of Short Report of the journal. We revised the statements according to the suggestion.

# Reviewer #3:

Prime editing, as a breakthrough in genome editing technology, is promising in realizing predefined, precise, and flexible editing both in animals and plants. However, low editing efficiency is by bar the bottleneck in plant prime editing. In this study, the authors achieved high prime editing efficiency in maize via enhancing pegRNA expression. Though the gene editing toolkit generated by Jiang et al seems promising, the manuscripts falls short of some major shortcomings listed below. A re-evaluation is necessary and the authors should submit a majorly revised version by addressing following comments.

Major comment:

(1) Experimental design of the manuscript is not very rigorous. For example, the P165S editing event was performed in both pZ1PE3 and pZ1PE3b vector, while unsuccessful desired edits were found in the transformation events. However, why not carry out the P165S editing event to compare the editing efficiency in pZ1WS vector. Moreover, the desired edits were just found in the transgenic lines transformed with pZ1WS, while not in the transgenic lines both transformed with pZ1PE3 and pZ1PE3b. Therefore, whether to generate the desired edits only need to use two promoter systems to drive pegRNA expression, together with the use of tRNA and ribozyme processing systems, without the need for additional pegRNA expression cassettes. Additionally, 16 transgenic lines are too few compared to the control.

## Response #3-1.

Thanks a lot. Actually, we constructed two optimized prime editors based on Csy4 system, p4xZ1PE3-Csy4 and p4xZ1PE3b-Csy4, and pZ1PE3 and pZ1PE3b were the controls of these two Csy4-based editors harboring redoubled pegRNA and sgRNA expression cassettes. However, the transformations of Csy4 vectors including these two were unexpectedly unsuccessful, which we attribute to the toxicity of Csy4 protein in maize. To clarify the doubt to the experimental design, we described these two plasmids in the revised main text. We designed the pZ1WS-Csy4 and pZ1WS editors for an attempt to generate herbicide-resistance maize lines rather than for comparison of editing efficiencies. This is the reason why these two editors lacked controls harboring single pegRNA cassettes. The pZ1WS vector was constructed as a control of pZ1WS-Csy4 for comparing the non-Csy4 system with the Csy4 system. We should have constructed more editors including controls especially after we achieved high editing efficiency with pZ1WS. However, maize transformation requires a long period (> 3 months) and too long delay of the publication will lead to lose the novelty. Testing in maize protoplasts seems a good idea, however, the maize protoplast transfection results are not always stable, especially when vectors have a large size (the size of pZ1WS is 24.7-kb). Therefore, we turned to testing in rice protoplasts and cooperated with Dr. Caixia Gao for the experiments. The results indicated: (1) the strategy based on CaMV35S-CmYLCV-U6 composite promoter significantly improved the prime-editing efficiencies for three of four pegRNAs; (2) the strategy based on redoubling the number of pegRNA cassettes had no contributions to improving prime-editing efficiency (Additional file 1: Fig. S3 and Table S4).

Yes, 16 transgenic lines are too few compared to the control, which is the reason why we repeatedly transformed maize with pZ1WS. Maize transformation is not easy and heavily dependent on availability of immature embryos. We foresaw that we could not generate many transgenic lines in our first transformation with pZ1WS, then we performed second transformation with pZ1WS after new batch of maize immature embryos were available. We added a sentence to the text to explain this.

(2) PE system produces various editing efficiencies at different target sites in previous studies. And this phenomenon is also reflected in Figure 2C. In my opinion, it is meaningless to perform two repeatedly transformation for plasmid pZ1WS targeting W542L and S621I in maize. Instead, it is proper to edit some other genome loci to further verify the efficiency of this system.

## Response #3-2.

Thanks a lot. Maize transformation is not easy and heavily dependent on availability of immature embryos. We foresaw that we could not generate many transgenic lines in our first transformation with pZ1WS, then we performed second transformation with pZ1WS after new batch of maize immature embryos were available. We added a sentence to the text to explain this. The pZ1WS vector was constructed as a control of pZ1WS-Csy4 for comparing the non-Csy4 system with the Csy4 system. Yes, we should have constructed more editors including controls especially after we achieved high editing efficiency with pZ1WS. We also agree that the more targets we tested, the higher quality of the results we could achieved. However, maize transformation requires a long period (> 3 months) and too long delay of the publication will lead to lose the novelty. Testing in maize protoplasts seems a good idea, however, the maize protoplast transfection results are not always stable, especially when vectors have a large size (the size of pZ1WS is 24.7-kb). Therefore, we turned to testing in rice protoplasts and cooperated with Dr. Caixia Gao for the experiments. The results indicated: (1) the strategy based on CaMV35S-CmYLCV-U6 composite promoter significantly improved the prime-editing efficiencies for three of four pegRNAs; (2) the strategy based on redoubling the number of pegRNA cassettes had no contributions to improving prime-editing efficiency (Additional file 1: Fig. S3 and Table S4).

(3) Among 129 plants detected, Li et al (2020, Mol Plant) achieved 15 individual homozygous plants carrying the precise edits. However, this is not mentioned in background. Instead, it mentions another example of inefficient editing. So in my opinion, this background is not comprehensive.

## Response #3-3.

Thanks a lot. Yes, Li et al. (2020) bombarded 148 calli, obtained 32 hygromycin-resistance calli, which produced 129 hygromycin-resistance plants including 15 homozygous mutations in the disrupted *hptII* gene conferring hygromycin-resistance if edits occur. However, these 15 individual homozygous plants were generated through the enriching strategy and the edited gene was not endogenous, which does not represent cases in practical applications. It is unfair to compare the editing efficiencies achieved by enriching strategies with those achieved in practical applications. That is the reason why we excluded the data based on enriching strategies.

Minor comment:

(1) In the Conclusion section, does the number of transgenic lines '62' mean the sum of two repeatedly transformation? If so, it is not reasonable because of the two independent experiments.

## Response #3-1b.

Thanks a lot. In our opinions, the more experiments are performed, the larger statistical population will be generated, and the higher quality of results can be anticipated.

(2) "For the W542L edits, the sgRNA nicking non-edited strand fully matches the ZmALS1 target", the description is incorrect. Nicking sgRNAs of PE3b with spacers match the edited strand, but not the original allele.

## Response #3-2b.

Thanks a lot. We revised the phase “the ZmALS1 target” to “the edited ZmALS1 target”.

(3) "In addition, we achieved two additional lines harboring the S621I homozygous mutations in the two ALS genes", the Sanger sequencing diagram of two S621I homozygous mutations lines should be provided.

## Response #3-3b.

Thanks a lot. We provided the sequencing chromatograms according to the suggestion (Additional file 1: Fig. S2).

(4) What does "double-strand even heteroduplex DNA repair" mean? Because no specific explanation is given in this article.

## Response #3-4b.

Thanks a lot. It means that the byproducts come from unbiased and well-balanced double-strand repair (rather than single-strand-biased repair) of multiple mismatches in heteroduplex DNA. This type of repair leads to incomplete editing of multiple nucleotides. We gave more explanation in the revised manuscript.

(5) The Figures looks confusing, further modification and improvement need to be done.

## Response #3-5b.

Thanks a lot. We improved the Figures as possible as we can.

**Second round of review**

**Reviewer 1**

The authors have addressed most of my questions and suggestions. But I have some minor comments on the revised manuscript.
1.     Page 3, Line 33-35. There are two major differences between pZ1WS and pZ1WS-Csy4 vector: 1, the pegRNA processing system (non-Cys4 with Cys4); 2, the promoter driving pegRNA expression (two promoters with single PolII promoter). Therefore, the pZ1WS vector was not an appropriate control of pZ1WS-Csy4.
2.     The prime editing efficiencies calculated in Figure 1c were based on Sanger sequencing results. However, according to the TA cloning results and NGS data, the editing frequencies at the W542L and S621I sites should be higher because the low-frequency mutations were detected. The authors should mention this difference in the figure or figure legend. I suggest the authors to use the NGS data to calculate the prime editing efficiencies because the NGS accurately detect all true editing events.
3.     Page 4, Line 44-46. Could the authors use a figure (or cite a reference) to help us understand the double-strand even DNA repair?
4.     In theory, both the CaMV35S-CmYLCV-U6 composite promoter and redoubling the number of pegRNA cassettes can enhance pegRNA expression and thus increase the prime editing efficiency. But the results from rice protoplasts showed that the CaMV35S-CmYLCV-U6 composite promoter significantly improved the prime editing efficiencies for three of four pegRNAs, whereas redoubling the number of pegRNA cassettes had no contributions to improving prime editing efficiency. The authors still could not give a reasonable explanation for the high editing efficiency of pZ1WS editor.

**Reviewer 2**

The authors have addressed my concerns in their revised manuscript, and I support publication.

**Reviewer 3**

In the revised version of the manuscript, the authors had added additional data and answered the questions that I raised. I do not have further questions.

**Authors Response**

**Point-by-point responses to the reviewers’ comments:**

Response to Reviewers

Reviewer #1: 1.Page 3, Line 33-35. There are two major differences between pZ1WS and pZ1WSCsy4 vector: 1, the pegRNA processing system (non-Cys4 with Cys4); 2, the promoter driving pegRNA expression (two promoters with single PolII promoter). Therefore, the pZ1WS vector was not an appropriate control of pZ1WS-Csy4.

*Response #1-1. Thanks a lot. To address this concern, we revised the sentence as follows: “The pZ1WS vector was constructed to use as a control of pZ1WS-Csy4 for comparing the non-Csy4 system with the Csy4 system”.*

2.The prime editing efficiencies calculated in Figure 1c were based on Sanger sequencing results. However, according to the TA cloning results and NGS data, the editing frequencies at the W542L and S621I sites should be higher because the lowfrequency mutations were detected. The authors should mention this difference in the figure or figure legend. I suggest the authors to use the NGS data to calculate the prime editing efficiencies because the NGS accurately detect all true editing events.

*Response #1-2. Thanks a lot. We added the values based on the NGS data to Figure 1c according to this suggestion.*

3.Page 4, Line 44-46. Could the authors use a figure (or cite a reference) to help us understand the double-strand even DNA repair?

*Response #1-3. Thanks a lot. We added schematic diagram to explain the double-strand even DNA repair-derived byproduct (Additional file 1: Fig. S1).*

4.In theory, both the CaMV35S-CmYLCV-U6 composite promoter and redoubling the number of pegRNA cassettes can enhance pegRNA expression and thus increase the prime editing efficiency. But the results from rice protoplasts showed that the CaMV35S-CmYLCV-U6 composite promoter significantly improved the prime editing efficiencies for three of four pegRNAs, whereas redoubling the number of pegRNA cassettes had no contributions to improving prime editing efficiency. The authors still could not give a reasonable explanation for the high editing efficiency of pZ1WS editor.

*Response #1-4. Thanks a lot. We discussed the reason as follows: “We attribute the unreasonable results from the redoubling strategy to low efficiency of the protoplast transfection since the large size (25.3-kb) of the vector very possibly had a negative effect on the protoplast transfection”.*
